# Environmental enrichment implies GAT-1 as a potential therapeutic target for stroke recovery

**DOI:** 10.7150/thno.53316

**Published:** 2021-01-27

**Authors:** Yuhui Lin, Mengcheng Yao, Haiyin Wu, Feng Wu, Shiying Cao, Huanyu Ni, Jian Dong, Di Yang, Yanyu Sun, Xiaolin Kou, Jun Li, Hui Xiao, Lei Chang, Jin Wu, Yan Liu, Chunxia Luo, Dongya Zhu

**Affiliations:** 1 Department of Pharmacology, School of Pharmacy, Nanjing Medical University, Nanjing 211166, China.; 2 Institution of Stem Cells and Neuroregeneration, Nanjing Medical University, Nanjing 211166, China.; 3 Department of Neurology, The Second Affiliated Hospital of Nanjing Medical University, Nanjing 210011, China.

**Keywords:** environmental enrichment, GAT-1, stroke, plasticity, functional recovery

## Abstract

**Rationale:** Stroke is a leading cause of adult disability worldwide, but no drug provides functional recovery during the repair phase. Accumulating evidence demonstrates that environmental enrichment (EE) promotes stroke recovery by enhancing network excitability. However, the complexities of utilizing EE in a clinical setting limit its translation.

**Methods:** We used multifaceted approaches combining electrophysiology, chemogenetics, optogenetics, and floxed mice in a mouse photothrombotic stroke model to reveal the key target of EE-mediated stroke recovery.

**Results:** EE reduced tonic gamma-aminobutyric acid (GABA) inhibition and facilitated phasic GABA inhibition in the peri-infarct cortex, thereby promoting network excitability and stroke recovery. These beneficial effects depended on GAT-1, a GABA transporter regulating both tonic and phasic GABA signaling, as EE positively regulated GAT-1 expression, trafficking, and function. Furthermore, GAT-1 was necessary for EE-induced network plasticity, including structural neuroplasticity, input synaptic strengthening in the peri-infarct cortex, output synaptic strengthening in the corticospinal tract, and sprouting of uninjured corticospinal axons across the midline into the territory of denervated spinal cord, and functional recovery from stroke. Moreover, restoration of GAT-1 function in the peri-infarct cortex by its overexpression showed similar beneficial effects on stroke recovery as EE exposure.

**Conclusion:** GAT-1 is a key molecular substrate of the effects of EE on network excitability and consequent stroke recovery and can serve as a novel therapeutic target for stroke treatment during the repair phase.

## Introduction

Stroke is a major cause of adult disability in much of the world 1, 2. Clinical treatments have improved in the acute time window, but long-term therapeutics remain limited to physical rehabilitation during the repair phase 1, 3. Accumulating evidence supports the concept that structural and functional remodeling of areas adjacent to the infarct core or remote regions can alter neuronal excitability within bilateral neuronal networks and thus contribute to functional recovery 4-7. Direct neuronal excitability enhancement approaches, including anodal direct current stimulation 8, transcranial magnetic stimulation 9, and optogenetic stimulation 10, have been shown to remap motor and sensory circuits, restore lost functions, and therefore improve the use of paretic limbs.

Environmental enrichment (EE) is known to profoundly remodel the central nervous system (CNS) at molecular, anatomical, and functional levels 11. By enhancing interactive social and physical activity, EE substantially modulates the network excitability of the rodent brain and is an effective nonpharmacologic and noninvasive approach to promote functional recovery from stroke 12, 13. Similar effects have also been noted in nonhuman primates 14 and humans 13, 15. While EE holds promise in its application as a therapeutic tool after stroke in humans, the complexities of utilizing EE in a clinical setting, including uncovered causal mechanisms, the difficulty in standardizing EE conditions, a lack of knowledge of active ingredients, and unknown required “dose”, make clinical translation difficult 13, 16. A good understanding of the molecular mechanisms underlying the effects of EE on network excitability and stroke recovery may identify pharmacological targets amenable to being artificially manipulated to mimic the impact of EE, thereby translating the essence of EE from the bench to the bedside for stroke treatment during the repair phase.

Functional recovery from stroke is related to gamma-aminobutyric acid (GABA) transporters (GATs). Four subtypes of GATs have been identified: GAT-1, -2, -3 and betaine GABA transporter. Of these, GAT-1 is the most abundantly expressed transporter in neurons, and the predominantly glial GAT-3 is also expressed in neurons 17, 18. Chronically elevated tonic inhibition caused by GATs dysfunction antagonizes the neuronal excitability required for functional recovery from stroke 5. In contrast, enhancing phasic inhibition promotes functional recovery from stroke 19. GAT-1 mediates this increase in phasic inhibition and decrease in tonic inhibition 20, 21. EE exposure substantially increases the density of the neurotransmitter gene co-expression network, in which *Gat-1* is a hub gene 22. Brain-derived neurotrophic factor (BDNF) has been proven to be crucial in EE-dependent stroke recovery 13, 23. BDNF effectively regulates the trafficking of GAT-1 that is crucial for its transporter function 17. In this study, we report that GAT-1 is crucial for EE-induced network plasticity and stroke recovery, and we reveal a novel pharmacological target for stroke treatment during the repair phase.

## Materials and methods

### Animals

*Slc6a1^flox/flox^*mice (GAT-1*^flox/flox^*) with exon 6 flanked by *loxP* recombination sites were generated at Shanghai Model Organisms following the generation methods described in detail in the following section. Wild-type male adult (6-7 weeks) C57BL6/J mice (research resource identifier, RRID: IMSR_JAX: 000664) were purchased from the Model Animal Research Center of Nanjing University. Wild-type C57BL6/J mice (embryonic day 16, male and female) were used for primary neuron cultures. Wild-type neonatal C57BL6/J mice (male and female) were used for primary astrocyte cultures. Animals were maintained at 20 ± 2 °C and under a 12 h light/dark cycle with food and water provided *ad libitum.* Every effort was made to minimize the number of animals used and their suffering. All experimental procedures were performed in accordance with protocols approved by the Institutional Animal Care and Use Committee of Nanjing Medical University (approval number: IACUC-1812012) and complied with the Animal Research: Reporting of *In vivo* Experiments (ARRIVE) guidelines.

### Generation of *Slc6a1^flox/flox^*mice

*Slc6a1^flox/flox^*(C57BL6/J background) mice were generated using the clustered regulatory inter spaced short palindromic repeats (CRISPR)/CRISPR-associated protein 9 (Cas9) (CRISPR/Cas9) system. A *Slc6a1* donor vector containing flox sites flanking exon 6 of the *Slc6a1* gene was created. Two single-guide RNAs (sgRNAs) targeting intron 5 and intron 6 were transcribed *in vitro*. The sgRNA target site for intron 5 was 5'-CGT GTA AAG AGT CTA GAG CC-3'. The sgRNA site for intron 6 was 5'-TAA GGT GCA CGG CAG ATG TT-3'. The donor vector with the two sgRNAs and Cas9 mRNA was microinjected into C57BL6/J fertilized eggs. F0 generation mice positive for homologous recombination were identified by polymerase chain reaction (PCR) analysis using two pairs of primers. A 4.3 kb fragment of the 5' end was amplified from the recombination allele with the primers P5-forward: 5'-GGG ATT GGG CCG TGA AGT TTT-3' and P5-reverse: 5'-CGG AAG TTG GGT GTG ATG TAG AAG-3'. A 3.9 kb fragment of the 3' end was amplified from the recombination allele with the primers P3-forward: 5'-AAC AAC GTG CTC CAG CGA CAA TGA-3' and P3-reverse: 5'-TGG CCA GGC AAG GAG AAA GACT-3'. The PCR products were further confirmed by sequencing.

Chimeras were mated with C57BL6/J mice to generate germline transmission. Heterozygous mice having the floxed *Slc6a1* allele were then bred to obtain homozygous mice. The genotype of this mouse line was confirmed by the pair of primers *Slc6a1^flox/flox^*(floxed allele) forward: 5'-GGA GGT GGC CTG GAA CAG C-3' and reverse: 5'-CTC CTG CCT GAG ACC TTG TAG TG-3'.

### Photothrombotic stroke model

Focal cortical strokes were induced in adult male mice through photothrombosis using a well-established procedure 24, 25. Under 2% isoflurane anesthesia (RWD Life Science), mice were fixed in a stereotaxic apparatus (Model 902, David Kopf Instruments) and their skulls were exposed through a midline incision, cleared of connective tissue, and dried. A cold light source (Z-LITE-Z, World Precision Instruments) attached to a custom-made opaque template giving a 2 mm diameter spot was precisely positioned 1.5 mm lateral from the bregma. Rose bengal solution (100 mg/kg, i.p.; Sigma-Aldrich) was then administered. After 5 min, the brain was illuminated through the exposed intact skull for 15 min. During illumination, rose bengal generated singlet oxygen leading to vascular endothelium damage and occlusion resulting in focal cortical stroke. Following illumination, the skin was surgically glued and the mice were allowed to recover. Control mice received the same surgery and the same dose of rose bengal but did not receive illumination. Core body temperature was measured by a rectal probe and maintained at 37 °C throughout the surgery.

### *In vivo* magnetic resonance imaging (MRI)

*In vivo* MRI was performed using a small animal MRI scanner (BioSpin, Bruker). Mice were anesthetized with 2% isoflurane. T2-TurboRARE imaging was conducted 1, 4, 12, 19, 26, and 33 days after stroke using a 2D fast-spin-echo sequence (repetition time/echo time: 3000 ms/33 ms; 1 average). Fourteen axial slices with a slice thickness of 1 mm, a matrix size of 256 × 256, and a field of view of 25 mm × 25 mm were positioned over the brain.

### Environmental enrichment (EE)

EE cages (90×70×30 cm^3^) were equipped with a variety of objects such as running wheels, tunnels, igloos, huts, retreats, and wooden toys. EE cages were fourteen times larger than standard housing (SH) cages (30×25×18 cm^3^). The objects in the EE cages were changed every other day to maintain a novel environment. Six mice were housed in each EE cage, while three mice were housed in each SH cage. Thus, the EE cage environment provided more opportunities for physical exercise, social interaction, and exposure to novel learning tasks compared with the SH cages. All mice received chow and water *ad libitum.*

### Recombinant virus production and stereotaxic injection

The adeno-associated virus (AAV) AAV-CaMKIIα-hM4D (Gi)-mCherry (5×10^12^ virus particles/mL) and AAV-VGAT-hM4D (Gi)-mCherry (3×10^12^ virus particles/mL) were purchased from BrainVTA. AAV-CaMKIIα-hChR2 (E123A)-eYFP (5×10^12^ virus particles/mL), AAV-hSyn-GAT-1-3Flag (7×10^12^ virus particles/mL), AAV-hSyn-GAT-1-3Flag-T2A-EGFP (7×10^12^ virus particles/mL), AAV-hSyn-3Flag-T2A-EGFP (7×10^12^ virus particles/mL), AAV-CAG-GAT-3-3Flag-T2A-mCherry (7×10^12^ virus particles/mL), AAV-CAG-3Flag-T2A-mCherry (7×10^12^ virus particles/mL) were purchased from GeneChem Co., Ltd. AAV-CAG-EGFP (7×10^12^ virus particles/mL) and AAV-CAG-EGFP-T2A-Cre (7×10^12^ virus particles/mL) were purchased from Obio Technology. Viruses were stored at -80 °C freezer until the day of infusion.

shRNAs of histone deacetylase (HDAC) 1 and 2 were constructed and synthesized by GeneChem Co., Ltd. The target sequence used against mouse *Hdac1* was 5'-GCC AGT CAT GTC CAA AGT AAT-3'. The target sequence used against mouse *Hdac2* was 5'-CAA TGA GTT GCC ATA TAA T-3'. Recombinant lentivirus expression plasmids and packaging plasmids were generated using Lipofectamine 2000. The specificity and efficiency of the shRNAs were validated and high titers of engineered lentiviruses (LV-Ubi-shHDAC1-GFP, 1 × 10^9^ virus particles/mL; LV-Ubi-shHDAC2-GFP, 1 × 10^9^ virus particles/mL) were produced, as described previously 26. LV-Ubi-GFP (1×10^9^ virus particles /mL) was used as control.

Six-week-old male C57BL6/J mice and GAT-1*^flox/flox^* mice were anesthetized with 2% isoflurane for stereotaxic viral injections. A small craniotomy was made targeting the motor cortex with stereotaxic coordinates (anteroposterior, 0 mm; mediolateral, -1.5 mm; dorsoventral, -1.3 mm; relative to bregma). Glass capillaries with a tip size of 30 μm in outer diameter were loaded with virus. Then, 500 nL of virus-containing solution was injected into the motor cortex at an injection rate of 1 nL/s. After injection, injection needles were left in place for an additional 10 min to assure even distribution of the virus and were then slowly withdrawn.

### Cannula implantation and drug microinjection

Immediately after photothrombotic stroke was induced, 26-gauge, 3.5 mm guide cannulae (RWD Life Sience) were implanted into the core of the infarction (anteroposterior: 0 mm; mediolateral: -1.5 mm; dorsoventral: -1.0 mm; relative to the bregma). The cannulae were fixed to the skull using adhesive luting cement and acrylic dental cement. Following surgery, stainless steel obturators were inserted into the guide cannulae to avoid obstruction until infusions were made. For drug infusions, obturators were removed and 33-gauge, 4.0 mm injectors (RWD Life Science) were placed into the guide cannulae. Injector tips extended 0.5 mm beyond the guide cannulae. During drug injection, 10 μM NO-711 (Tocris Bioscience), or 1 mg/mL TrkB-Fc (R&D system) were slowly infused at a flow rate of 0.2 μL/min to a total volume of 2 μL. Following infusion, the injectors were left in place for an additional 5 min to allow for diffusion of the drug. Following microinjection, the stainless steel obturators were subsequently reinserted into the guide cannulae.

### Behavioral experiments

Mice were tested by grid-walking tasks and spontaneous forelimb (cylinder) tasks 7 days before surgery to establish baseline performance and subsequently 4, 12, 19, 26, and 33 days after stroke to evaluate functional deficits. The mice were identified by earmarks and numbered only after finishing the behavioral experiments.

Grid-walking tasks were performed as previously described in detail with minor modifications 24. Briefly, each mouse was placed individually on top of an elevated wire grid (length, 32 cm; width, 20 cm; height, 50 cm) composed of 12 mm square wire meshes and allowed to walk freely for 5 min. A video camera (C525, Logitech) was positioned 30 cm beneath the wire grid to capture stepping errors (foot faults). The video footage was analyzed offline in slow motion (1/5th real time speed) by a rater who was blind to the treatment groups. If a step did not provide support and the foot went through the grid it was considered a fault. A step was also considered a foot fault if the mouse was resting with the grid at the level of the wrist. The total number of steps for each limb was counted and the ratio between foot fault steps and total steps was calculated. Differences in the degree of locomotion between animals and trials were accounted for by calculating the ratio between foot faults and total steps taken.

Cylinder tasks were performed as previously described in detail with minor modifications 24. Briefly, each mouse was placed individually in a clear plexiglass cylinder (height, 15 cm; diameter, 10 cm). The mouse would spontaneously rear to a standing position while supporting its weight with either one or both of its forelimbs. A video camera (C525, Logitech) was positioned 20 cm in front of a cylinder to record the free exploration of the mouse for 5 min. The video footage was analyzed offline in slow motion (1/5^th^ real time speed) by calculating the time (s) during each rear that the animal spent on either its right forelimb, left forelimb, or both forelimbs. Only rears in which both forelimbs could be clearly seen were included. The percentage of time spent on each limb was calculated and these data were used to derive an asymmetry index as follows: (% ipsilateral use) - (% contralateral use).

### Infarct size measurement

Infarct size was measured by Nissl staining according to the manufacturer's protocol (Beyotime Biotechnology). Briefly, coronal slices (40 μm) were cut using a vibrating blade microtome (VT1200s, Leica). Every 8^th^ section throughout the infarct area was collected and processed for Nissl staining. The sections were immersed in Nissl staining solution for 10 min at 40 °C. Images were captured with a microscope (Axio, Carl Zeiss).

### *In vitro* studies

Human cortical neurons were differentiated from human embryonic stem cells (hESCs; H9, passages 60-80, WiCell Agreement No. 16-W0060) according to the following protocol. hESCs were detached with 1 U/mL Dispase (Thermo Fisher Scientific) to form embryoid bodies in suspension in neural induction medium consisting of DMEM/F12 (Gibco), N2 supplement (1:100; Gibco), and non-essential amino acids (1:100; Gibco) for 7 days. Embryoid bodies were attached on day 7 and neural tube-like rosette structures were formed on days 10-16. Rosette colonies were detached by gently blowing with a pipette and suspended to form neural spheres the next day. For neuronal differentiation, the neural spheres were dissociated with TrypLE (Thermo Fisher Scientific) and attached onto a coverslip precoated with poly-L-ornithine (Sigma-Aldrich). The medium was refreshed every 5-6 days.

Primary neurons were isolated from E16 wild-type mice cortex and cultured on dishes coated with poly-L-ornithine in neurobasal medium (Gibco) containing 2% B27 supplement (Gibco), as reported previously 27. Cortical neurons were plated at a density of 1×10^4^ cells/cm^2^ and infected with LV-GFP for morphological analysis.

Primary astrocytes were isolated from neonatal wild-type mice cortex and cultured on dishes coated with poly-L-ornithine in DMEM/F12 medium containing 10% fetal calf serum (Gibco), as reported previously 27.

To mimic the *in vivo* conditions of ischemia, hESCs-differentiated cortical neurons (43-45 days *in vitro*, DIV), primary mouse cortical cultures (10-12 DIV), and primary mouse astrocyte cultures were rinsed twice with serum- and glucose-free medium and incubated with serum- and glucose-free medium in a hypoxia chamber (C21, Biospherix). The chamber was flushed for 3 h with N_2_ and CO_2_ to maintain 2% O_2_ and 5% CO_2_. After oxygen glucose deprivation (OGD), the serum- and glucose-free medium was replaced with normal medium and the cultures were returned to a normoxic incubator. The cultures were treated with 0.5 μM trichostatin A (TSA; Selleck Chemicals) or a mixture of 0.5 μM TSA and 10 μM NO-711 (Tocris Bioscience) 24 h after OGD. An equal volume of vehicle was added as control. After 24 h of drug incubation, the cultures were fixed with 4% paraformaldehyde (PFA) and labeled for immunostaining. Astrocytes were harvested for Western blot analysis at 24, 48, and 72 h after TSA treatment. All cultures were maintained in an incubator (HERAcell 150, Thermo Fisher Scientific) with a humidified atmosphere of 95% air and 5% CO_2_ at 37 °C, except during OGD.

### Extraction of membrane protein fractions

Membrane protein fractions were extracted using a kit (Merck) according to the manufacturer's protocol. Briefly, peri-infarct tissue or corresponding tissue of sham-operated mice was dissected and homogenized until intact pieces were no longer visible. The homogenized protein mixture was incubated with gentle agitation then centrifugated at 1000 ×*g* for 5 min to separate cytosol and membrane protein fractions. Phase partitioning resulted in the hydrophilic cytosol proteins layering at the top and the hydrophobic membrane proteins at the bottom. The isolated membrane and cytosol proteins were detected by immunoblotting. To test the purity of the isolated proteins, we measured β-actin (a cytosol protein) and E-cadherin (a membrane protein) by western blot.

### Western blot

Western blot was performed as described in detail previously 28. Briefly, peri-infarct cortex tissue was rapidly dissected over an ice box as previously described 29. The corresponding cortex region of sham-operated mice was also dissected. The following primary antibodies were used: rabbit anti-histone H4 (1:500; Abcam, RRID: AB_29688), rabbit anti-acetylated histone H4 (acetyl-K5) (1:1000; Abcam, RRID: AB_2264109), rabbit anti-GAT-1 (1:2000; Abcam, RRID: AB_2189971), rabbit anti-GAT-3 (1:1000; Abcam, RRID: AB_304437), rabbit anti-HDAC1 (1:2000; Abcam, RRID: AB_470299), rabbit anti-HDAC2 (1:2000; Abcam, RRID: AB_732777). Mouse anti-E-cadherin (1:4000; Thermo Fisher Scientific, RRID: AB_2533118), mouse anti-GAPDH (1:4000; Kangchen Biotech, RRID: AB_2493106) and mouse anti-β-actin (1:4000; Sigma-Aldrich, RRID: AB_476692) were used as internal references. Secondary antibodies including HRP-linked goat-anti-rabbit and goat-anti-mouse antibodies were used for detection by enhanced chemiluminescence (Millipore). All experiments were performed at least three times.

### Immunostaining

The detailed methods for immunofluorescence of brain sections and cultured cells were described in detail previously 27. In brief, for immunohistochemistry, mice were transcardially perfused with saline followed by 4% PFA under deep chloral hydrate anesthesia (400 mg/kg, i.p.; HuShi). Brains were removed, post-fixed overnight, and then coronal sections (40 μm) were cut on a vibratome (VT1200s, Leica). For immunocytochemistry, cells were fixed using 4% PFA. The Slices were blocked in phosphate-buffered saline containing 3% normal goat serum, 0.3% (w/v) Triton X-100, and 0.1% bovine serum albumin at room temperature for 1 h, then incubated in primary antibody at 4 °C overnight. For CaMKⅡ immunostaining, sections were subjected to antigen retrieval with sodium citrate (pH 7.0) at 60 °C for 2 h. The primary antibodies used were as follows: mouse anti-CaMKⅡ (1:50; CST, RRID: AB_2721906), rabbit anti-acetylated histone H4 (acetyl-K5) (1:300; Abcam, RRID: AB_2264109), mouse anti-Tuj-1 (1:500; Sigma-Aldrich, RRID: AB_477590), rabbit anti-GAT-1 (1:800; Synaptic Systems, RRID: AB_2620000), mouse anti-NeuN (1:500; Millipore, RRID: AB_2298772), rabbit anti-synapsin (1:400; Millipore, RRID: AB_90757), mouse anti-PSD-95 (1:200; Abcam, RRID: AB_303248), mouse anti-Flag (1:500; Sigma-Aldrich, RRID: AB_262044) and mouse anti-GFAP (1:1000; Millipore, RRID:AB_11212597). The secondary antibodies used were as follows: goat anti-mouse Cy3 (1:200; Jackson ImmunoResearch Laboratories, RRID: AB_2338680), goat-anti mouse Alexa-488 (1:400; Jackson ImmunoResearch Laboratories, RRID: AB_2338840), goat anti-rabbit Cy3 (1:200; Jackson ImmunoResearch Laboratories, RRID: AB_2338000), and goat anti-rabbit Alexa-488 (1:400; Jackson ImmunoResearch Laboratories, RRID: AB_2338046). For the biocytin-injected slices streptavidin-Cy3 (1:1000; Sigma-Aldrich) was added to the secondary antibody solution. Finally, Hoechst 33258 (Sigma-Aldrich) was used to counterstain the slices or cultures to label the nuclei. Images were captured with a confocal laser-scanning microscope (LSM700, Carl Zeiss) at identical settings for each condition.

### Chromatin immunoprecipitation (ChIP)

ChIP assays were performed using a kit (EMD Millipore) according to the manufacturer's protocol. Briefly, peri-infarct tissue or corresponding tissue of sham-operated mice was homogenized, chemically cross-linked in 1% formaldehyde for 10 min, and then quenched with 125 mM glycine for 5 min. Chromatin was sheared to 200-1000 bp using a sonifier (Branson Digital Sonifier 450) set to 45% maximum amplitude for twenty-five 20 s pulses with 50 s pauses. The samples were immersed in an ice bath during shearing. After centrifugation, cell debris was removed and the supernatants were diluted with ChIP dilution buffer. A fraction of the diluted supernatant was collected as immunoprecipitation input control. The remainder was incubated with Protein A-Sepharose beads for 30 min at 4 °C to reduce nonspecific background. The beads were removed by centrifugation, and the immunoprecipitating antibody against acetylated histone H4 (Abcam, RRID: AB_2264109) was added to the supernatant and incubated overnight. Then, immune complexes were enriched by incubation with Protein A-Sepharose beads for 1 h at 4 °C. The beads were collected and subjected to a series of sequential washes. Chromatin was eluted from the beads by agitation in elution buffer containing 1% sodium dodecyl sulfate and 0.1 M NaHCO_3_, and crosslinking was reversed by incubating the samples overnight at 65 °C. Purified DNA samples were normalized and subjected to real-time PCR for 45 cycles, using primer pairs specific to 150-250 bp segments corresponding to mouse gene promoter regions (regions upstream of the start codon, near the first exon).

### Real-time PCR

Real-time PCR was performed with TB-Green reagents (TaKaRa) using a real-time PCR detection system (Lightcycler96, Roche). The relative quantities of immunoprecipitated DNA fragments were calculated by the ΔΔC_T_ method. All reactions were performed in triplicate.

The primer sequences used for PCR were *Gat-1* forward: CAT GGG TGG ACC TAT GCC TC, *Gat-1* reverse: AGG GAC ACA ATG GTA GAC GC; *Gat-3* forward: CTG CAT GGT GCA TGG TCA TTG, *Gat-3* reverse: GCT GAG TAT CCT GCT TCA CCT;* Bdnf-p1* forward: TGA TCA TCA CTC ACG ACC ACG, *Bdnf-p1* reverse: CAG CCT CTC TGA GCC AGT TAC G; *Bdnf-p4* forward: GCG CGG AAT TCT GAT TCT GGT AAT, *Bdnf-p4* reverse, GAG AGG GCT CCA CGC TGC CTT GAC G.

### Slice preparation

Under deep ethyl ether anesthesia, mice were decapitated and their brains and spinal cords were quickly removed. Slices (thickness, 350 μm) were cut in ice-cold cutting solution containing 110 mM choline chloride, 20 mM glucose, 2.5 mM KCl, 0.5 mM CaCl_2_, 7 mM MgCl_2_, 1.3 mM NaH_2_PO_4_, 25 mM NaHCO_3_, 1.3 mM sodium ascorbate, and 0.6 mM sodium pyruvate (pH 7.4). The slices were transferred to an interface-style chamber and incubated in artificial cerebrospinal fluid (ACSF) containing 10 mM glucose, 125 mM NaCl, 2.5 mM KCl, 2 mM CaCl_2_, 1.3 mM MgCl_2_, 1.3 mM NaH_2_PO_4_, 25 mM NaHCO_3_, 1.3 mM sodium ascorbate, and 0.6 mM sodium pyruvate. The slices were recovered at 34 °C for at least 1 h before recording. All external solutions were saturated with 95% O _2_ / 5% CO_2_ gas.

### Whole-cell recordings

Slices or hESCs-differentiated neurons (43-45 DIV) were transferred to a recording chamber that was continuously perfused with oxygenated ACSF (flow rate, 4-6 mL/min). The slices were visualized using an upright microscope (Olympus X51W, Nomasky) with a 5× 0.1 NA or 60× 1.0 NA objective and infrared and differential interference contrast optics. The fluorescent neurons were visualized under an Olympus X51W microscope equipped with a 60× water-immersion lens and illuminated with a mercury lamp. Whole-cell recordings were made in pyramidal neurons from peri-infarct tissues or tissues from corresponding locations in sham-operated mice using patch-clamp electrodes (tip resistance, 6-8 MΩ). All recordings were low-pass filtered at 2 kHz and sampled at 10 kHz using a digitizer (Digidata 1440A, Axon Instruments). Access resistance was 5-25 MΩ and was monitored throughout the experiment (neurons with access resistance changes > 20% were excluded). Data were collected with pClamp 10.3 software and analysed using Clamfit 10.3 (Molecular Devices). All experiments were performed by a researcher blinded to group allocation.

To reconstruct the morphology of the putative pyramidal neurons, 0.2% biocytin (Sigma-Aldrich) was added to the internal solution. After recording, the micropipette was gently retracted from the cell to achieve an outside-out patch for better morphological reconstruction of biocytin-filled neurons. Slices were fixed with 4% PFA and processed for post hoc immunohistochemistry.

For tonic inhibitory current (*I*_tonic_) and mean phasic current recordings, patch-clamp electrodes were filled with an intracellular solution containing 120 mM CsMeSO4, 10 mM CsCl, 5 mM TEA-Cl, 1.5 mM MgCl_2_, 10 mM HEPES, 0.1 mM EGTA, 2 mM Na-ATP, 0.5 mM Na-GTP, and 5 mM QX-314 with the pH adjusted to 7.25-7.30 by CsOH (275-285 mOsmol). To replenish the extracellular GABA concentration reduced by the high-flow perfusion of the slices, the recording ACSF was supplemented with 5 μM GABA. *I*_tonic_ and mean phasic current were recorded by voltage clamping at +10 mV using an amplifier (Axonpatch-700B, Axon Instruments). *I*_tonic_ was recorded as the baseline shift after administration of 100 μM bicuculline methiodide (BMI; Tocris Bioscience). Tonic current density was calculated as follows: current amplitude/membrane capacitance. The amplitude and frequency of spontaneous inhibitory post-synaptic current (sIPSC) before BMI administration were analyzed using MiniAnalysis Program 6.0 (Synaptosoft Inc.).

For action potential (AP) recordings, glass pipettes were loaded with an intracellular solution containing 70 mM potassium gluconate, 70 mM KCl, 2 mM NaCl, 2 mM MgCl_2_, 10 mM HEPES, 1 mM EGTA, 2 mM MgATP, and 0.3 mM Na_2_GTP with the pH adjusted to 7.25-7.30 by KOH (275-285 mOsmol).

For spontaneous postsynaptic current (sPSC) recordings in hESCs-differentiated neurons, the membrane potential was held at -70 mV with the following intracellular solution: 140 mM CsF, 10 mM BAPTA, 1.0 mM CaCl_2_, 2 mM MgCl_2_, 10 mM HEPES, and 4 mM K_2_ATP_4_ (pH 7.25-7.30).

For miniature excitatory postsynaptic current (mEPSC) recordings, microelectrodes were filled with internal pipette solution, containing 132.5 mM cesium gluconate, 17.5 mM CsCl, 2 mM MgCl_2_, 0.5 mM EGTA, 10 mM HEPES, 4 mM ATP, 5 mM QX-314 with the pH adjusted to 7.25-7.30 by CsOH (275-285 mOsmol). To study mEPSC activity in isolation, 0.5 μM tetrodotoxin and 20 μM BMI were added to block action potentials and GABA_A_ receptor-mediated currents, respectively. The recordings were longer than 5 min. Data were analyzed using MiniAnalysis Program 6.0 (Synaptosoft Inc.). Up to 100 events from each neuron were selected at a fixed sampling interval to generate cumulative probabilities.

For channelrhodopsin-2 (ChR2)-mediated current recordings, microelectrodes were filled with internal pipette solution containing 132.5 mM cesium gluconate, 17.5 mM CsCl, 2 mM MgCl_2_, 0.5 mM EGTA, 10 mM HEPES, 4 mM ATP, and 5 mM QX-314 with the pH adjusted to 7.25-7.30 by CsOH (275-285 mOsmol). We added 10 μM NBQX and 20 μM BMI to block AMPA receptor-mediated currents and GABA_A_ receptor-mediated currents, respectively. Optogenetic stimulation during recordings was performed using a 470 nm light-emitting diode (LED) source coupled to a fiber guide and a collimator lens to focus the light spot around the recorded neuron.

### Local field potential (LFP) recordings

For LFP recordings, microelectrodes (1-2 MΩ) were filled with ACSF. A collimated 470 nm LED was used to photostimulate ChR2-expressing peri-infarct motor cortex fibers in the C5-C7 cervical spinal cord segment. The slices were stimulated by a single 20 ms light pulse repeated every 4 s for 20 repetitions.

### Tract-tracer injection and quantification of axonal sprouting

Tract tracing was used to assess post-stroke axonal sprouting, as described previously 30. On day 34 after photothrombotic stroke, mice were anesthetized with 2% isoflurane and fixed in a stereotaxic apparatus with the skull exposed. The tract tracer biotinylated dextran amine (BDA; Molecular Probes) was dissolved in phosphate-buffered saline at a concentration of 10% and 2 µL was injected at two sites in the contralesional cortex: (anteroposterior: 0.6 mm, mediolateral: 1.2 mm, dorsoventral: -1.5 mm; anteroposterior: 0 mm, mediolateral: 1.8 mm, dorsoventral: -1.7 mm; relative to bregma anteroposterior). Brains and spinal cords were harvested 14 days post injection after cardiac perfusion with saline followed by 4% PFA. Coronal brain sections and transverse cervical spinal cord sections were cut at a thickness of 40 µm. BDA was visualized by treatment with streptavidin-Cy3 (1:500; Jackson Immuno-Research Laboratories, RRID: AB_2337244). The number of midline-crossing BDA^+^ fibers (from intact to lesioned side) was manually counted by an investigator blinded to group allocation. Three sections (80 µm apart) at the level of the medullary pyramids from each mouse were assessed.

### Randomization, inclusion and exclusion criteria

All mice were numbered by earmarks and were randomly divided into groups using a table of random numbers generated using Microsoft Excel. Modified neurological severity score, which is positively correlated with infarct volume, was tested 3 days after stroke to assess neurological outcome 26. Neuroscore was graded from 0 to 18 (normal score, 0; maximal deficit score, 18). Severe injury was indicated by a score of 13-18, moderate injury as, 7-12, and mild injury as, 1-6. Mice whose neuroscore was < 7 (with mild infarct size) were excluded. For recombinant virus experiments, mice were randomly allocated to groups using computer-generated random numbers before stroke and mice with neuroscores < 7 were excluded at 3 days after stroke. After completion of the behavioral tests, the mice were identified by earmarkers and were given their numbers. Then, mouse brains were harvested under deep anesthesia (400 mg/kg, i.p., chloral hydrate) for confirmation of viral infection. To validate specificity, sensitivity, and the spatial distribution of viral infection, brain slices were prepared for optical microscopy and immunohistochemistry. Coronal sections were stained for Hoechst. If the GFP- or mCherry-immunoreactivity within the peri-infarct area (within 400 μm of the infarct) was >80%, and the GFP- or mCherry-immunoreactivity outside the peri-infarct area was <10%, then the mouse was considered effectively infected. Animals were excluded from the analyses if their peri-infarct areas were not effectively infected. No animals were replaced after death or exclusion.

### Statistical analyses

Data are presented as mean ± standard error of the mean (SEM). Comparisons among multiple groups in the behavioral assessments were made with two-way repeated-measures ANOVA followed by post hoc Bonferroni test (SPSS Statistics 22 software), and other comparisons among multiple groupswere made with one-way ANOVA followed by post hoc Scheffe test (Stata 9.0 software). Comparisons between two groups were made with two-tailed student's *t*-test (Stata 9.0 software). The threshold level of significance was set at *p* < 0.05. The sample size was obtained with power analysis and sample size software using a significance level of α = 0.05 with 90% power to detect statistical differences. For animal studies, the sample size was predetermined by our prior experiments. All experiments and data analyses were conducted blinded.

## Results

### EE restores neuronal excitability and GAT-1 function in the peri-infarct cortex

**Figure 1:**
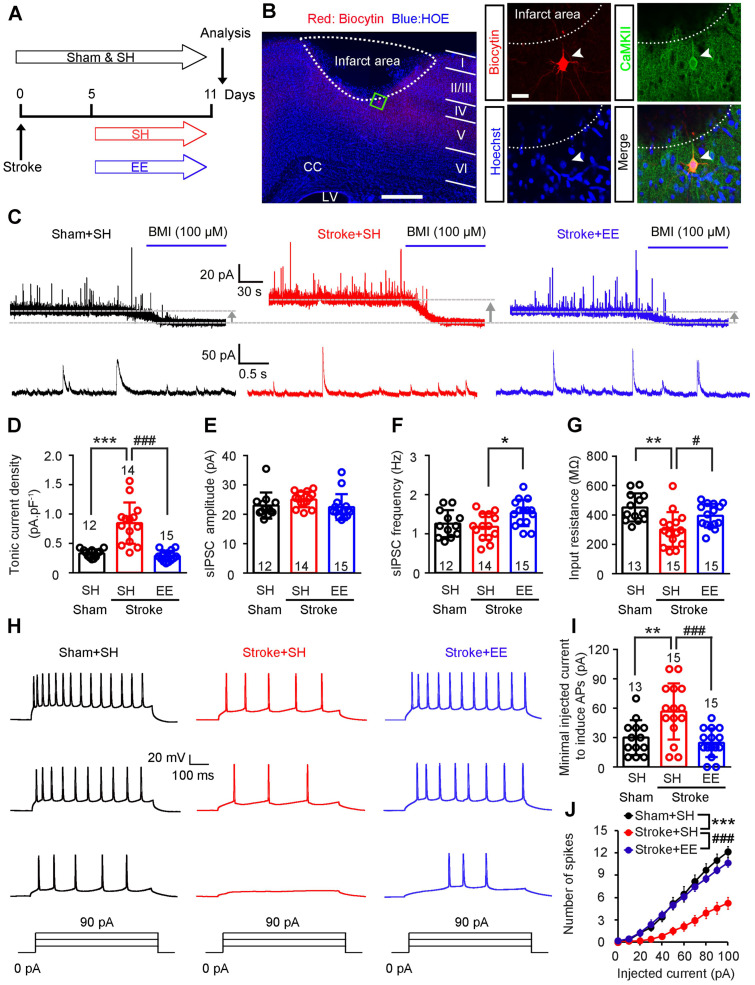
** EE restores neuronal excitability by reducing tonic and facilitating phasic GABA inhibition in the peri-infarct cortex.** (A) Experimental design. (B) A putative layer 5 pyramidal neuron of the peri-infarct area (within 400 µm of the infarct) recorded using a biocytin-containing solution and co-labeled with CaMKII. Left scale bar, 500 µm. Right scale bar, 20 µm. (C) Top, representative traces showing tonic inhibitory currents recorded from layer 5 pyramidal neurons in Sham + SH, Stroke + SH, and Stroke + EE mice. Dashed lines and arrows indicate baseline shift after blocking all GABA_A_ receptors with BMI (100 µM). Bottom, representative traces showing phasic inhibitory currents recorded from layer 5 pyramidal neurons in Sham + SH, Stroke + SH and Stroke + EE mice. (D) Bar graph showing tonic current density from layer 5 pyramidal neurons in the peri-infarct cortex 12 days after stroke. One-way ANOVA followed by post hoc Scheffe test, *F*_(2,38)_ = 28.86, ^***^p < 0.001, ^###^*p* < 0.001. (E) Bar graph showing the amplitude of sIPSCs in the peri-infarct cortex 12 days after stroke. One-way ANOVA followed by post hoc Scheffe test, *F*_ (2,38)_ = 1.71. (F) Bar graph showing the frequency of sIPSCs in the peri-infarct cortex 12 days after stroke. One-way ANOVA followed by post hoc Scheffe test, *F*_(2,38)_ = 4.36, ^*^p = 0.027. (G) Bar graph showing the input resistance of peri-infarct pyramidal neurons 12 days after stroke. One-way ANOVA followed by post hoc Scheffe test, *F*_(2,40)_ = 8.19, ^**^p = 0.001, ^#^*p* =0.046. (H) Representative APs in peri-infarct pyramidal neurons evoked by current injections from 0 to 90 pA in 30 pA steps. (I) Bar graph showing the minimal injected current to induce APs in peri-infarct pyramidal neurons. One-way ANOVA followed by post hoc Scheffe test, *F*_(2,40)_ = 9.52, ^**^p = 0.008, ^###^*p* < 0.001. (J) Number of APs in peri-infarct pyramidal neurons evoked by various current steps. Two-way repeated-measures ANOVA followed by post hoc Bonferroni test, *F*_(2,40)_ = 13.227, ^***^p < 0.001, ^###^*p* < 0.001. n = 13 neurons (Sham+SH), 15 neurons (Stroke+SH), and 15 neurons (Stroke+EE) from 5-6 mice. AP, action potential; BMI, bicuculline methiodide; CC, corpus callosum; EE, environmental enrichment; GABA, gamma-aminobutyric acid; LV, lateral ventricle; SH, standard housing; sIPSC, spontaneous inhibitory postsynaptic current.

**Figure 2:**
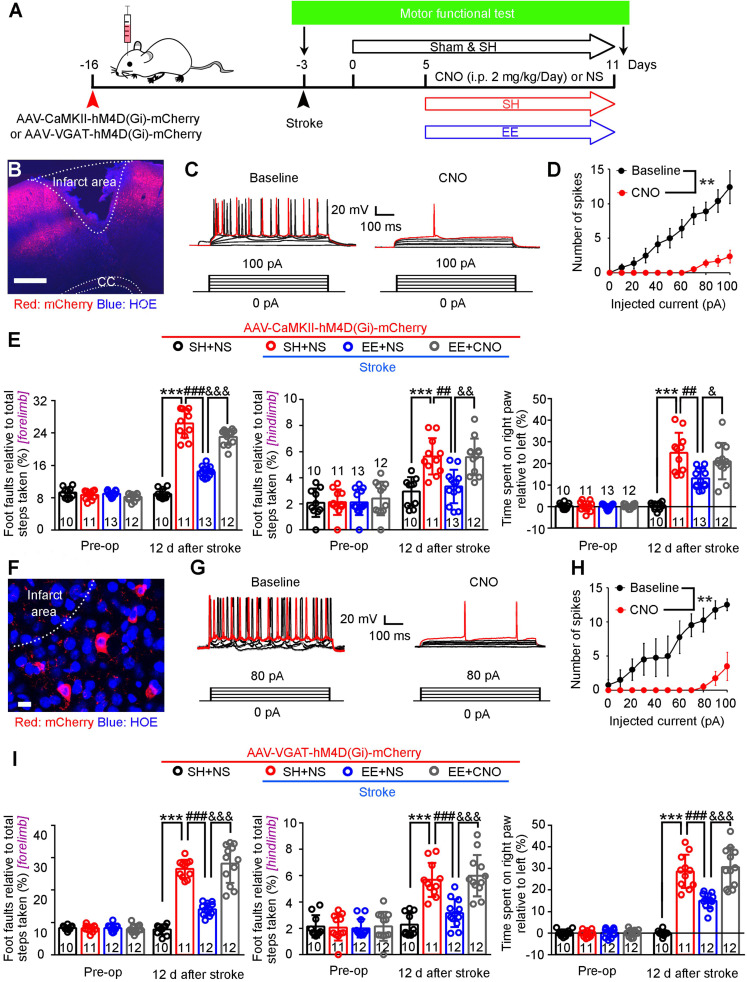
** Silencing the activity of principle neurons or interneurons abolishes the effect of EE on stroke recovery.** (A) Diagram showing the infusion of AAV-CaMKII-hM4D(Gi)-mCherry or AAV-VGAT-hM4D(Gi)-mCherry into mouse motor cortex and the experimental design for (B-I). (B) Representative image of peri-infarct cortex infected with AAV-CaMKII-hM4D(Gi)-mCherry on day 5 after stroke. Scale bar, 500 µm. (C) Current-voltage relationship of a representative peri-infarct virus-infected neuron recorded before and during CNO (5 µM) perfusion. Raw traces showing individual voltage responses to a series of 600 ms current pulses from 0 to 100 pA in 20 pA steps. Red traces indicate the number of 100 pA current-induced APs. (D) Number of induced APs in peri-infarct pyramidal neurons at various current steps (n = 8 neurons from 5 animals for each group). Two-way repeated-measures ANOVA, *F*_(1,14)_ = 18.092, ^**^p = 0.001. (E) Left, foot faults of the left forelimb in the grid-walking task. Middle, foot faults of the left hindlimb in the grid-walking task. Right, forelimb symmetry in the cylinder task. One-way ANOVA followed by post hoc Scheffe test. Left, *F*_(3,42)_ = 143.98, ^***^p < 0.001, ^###^*p* < 0.001, ^&&&^*p* < 0.001. Middle, *F*_(3,42)_ = 13.72, ^***^p < 0.001, ^##^*p* = 0.001, ^&&^*p* = 0.001. Right, *F*_(3,42)_ = 27.71, ^***^p < 0.001, ^##^*p* = 0.001, ^&^*p* = 0.046. (F) Representative image showing peri-infarct cortex infected with AAV-VGAT-hM4D(Gi)-mCherry on day 5 after stroke. Scale bar, 20 µm. (G) Current-voltage relationship of a representative peri-infarct virus-infected neuron recorded before and during CNO (5 µM) perfusion. Raw traces showing individual voltage responses to a series of 600 ms current pulses from 0 to 100 pA in 20 pA steps. Red traces indicate the number of 100 pA current-induced APs. (H) Number of induced APs in peri-infarct interneurons at various current steps (n = 4 neurons from 3 animals for each group). Two-way repeated-measures ANOVA, *F*_(1,6)_ = 14.512. ^**^p = 0.009. (I) Left, foot faults of the left forelimb in the grid-walking task. Middle, foot faults of the left hindlimb in the grid-walking task. Right, forelimb symmetry in the cylinder task. One-way ANOVA followed by post hoc Scheffe test. Left, *F*_(3,41)_ = 80.6, ^***^p < 0.001, ^###^*p* < 0.001, ^&&&^*p* < 0.001. Middle, *F*_(3,41)_ = 24.06, ^***^p < 0.001, ^###^*p* < 0.001, ^&&&^*p* < 0.001. Right, *F*_(3,41)_ = 52.71, ^***^p < 0.001, ^###^*p* < 0.001, ^&&&^*p* < 0.001. AP, action potential; CC, corpus callosum; CNO, clozapine-*N*-oxide; EE, environmental enrichment; SH, standard housing.

To determine whether tonic and phasic GABA inhibition are implicated in EE-induced neuronal excitability in the peri-infarct zone during the repair phase of stoke, we induced photothrombotic stroke in the forelimb motor cortex of mice, in which the ischemic infarct core and peri-infarct zone were well defined 26. We exposed mice to EE or SH on days 5-11 and performed electrophysiology on day 12 after stroke (**Figure 1A**). Peri-infarct pyramidal neurons were recorded in whole-cell mode and identified using biocytin (**Figure 1B**). Whole-cell voltage-clamp recordings in acute brain slices showed that EE blocked the stroke-induced increase in GABA_A_R-mediated *I*_tonic_ in layer 5 pyramidal neurons (**Figure 1C-D**). Additionally, EE significantly increased sIPSC frequency but not amplitude compared to SH (**Figure 1C**,** E-F**), suggesting an enhanced phasic GABA inhibition through presynaptic mechanisms. Tonic inhibition in layer 5 neurons in the neocortex plays a critical role in the control of network excitability 31. We thus examined whether EE restores neuronal excitability in the peri-infarct cortex. We directly detected the firing properties of peri-infarct pyramidal neurons in whole-cell current-clamp mode and found that EE reversed stroke-induced decreases in input resistance and sensitivity to depolarizing current injections (**Figure 1G-J**), indicating an EE-induced excitability enhancement. The EE-induced excitability enhancement of principle neurons in the peri-infarct cortex is necessary for stroke recovery (**Figure 2A-E**). Moreover, we investigated whether the excitability of inhibitory interneurons is implicated in EE-induced stroke recovery and found that silencing the activity of peri-infarct interneurons also cancelled the beneficial effects of EE on stroke recovery (**Figure 2A**, **F-I**), suggesting the importance of phasic GABA signaling in EE-mediated stroke recovery. Additionally, EE exposure during the repair phase of stroke did not affect infarct volume (**Figure S1A-B**).

**Figure 3:**
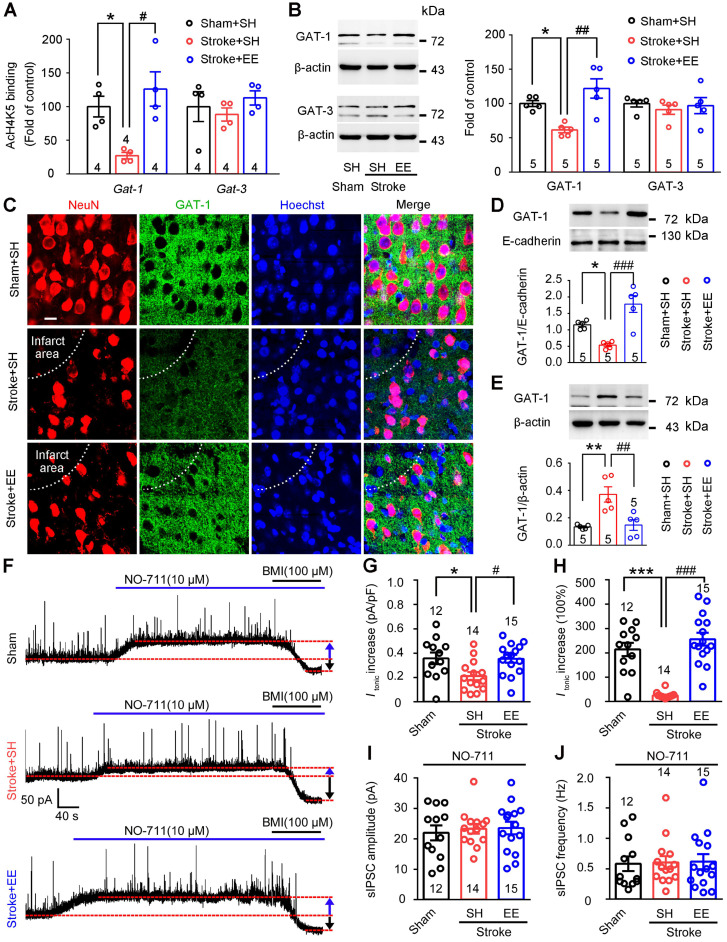
** EE restores stroke-induced GAT-1 downregulation and dysfunction in the peri-infarct cortex.** (A) Bar graph showing changes in histone acetylation of the promoter regions of GATs genes in peri-infarct tissue. Fragmented chromatin was immunoprecipitated with antibody recognizing acetyl-H4 and quantified with real-time polymerase chain reaction. One-way ANOVA followed by post hoc Scheffe test. For *Gat-1*, *F*_(2,9)_ = 8.74, ^*^p = 0.046, ^#^*p* = 0.01; for *Gat-3*, *F*_(2,9)_ = 0.66. (B) Representative immunoblots and bar graph showing levels of GATs proteins in the peri-infarct cortex. For GAT-1, *F*_(2,12)_ = 11.9, ^*^p = 0.031, ^##^*p* = 0.002; for GAT-3, *F*_(2,12)_ = 0.3. (C) Representative fluorescence image showing NeuN (a mature neuron marker) and GAT-1 in the peri-infarct cortex. Scale bar, 20 μm. (D) Representative immunoblots and bar graph showing GAT-1 content in membrane fractions. One-way ANOVA followed by post hoc Scheffe test, *F*_(2,12)_ = 16.79, ^*^p = 0.044, ^###^*p* < 0.001. (E) Representative immunoblots and bar graph showing GAT-1 content in cytosol fractions. One-way ANOVA followed by post hoc Scheffe test, *F*_(2,12)_ = 11.49, ^**^p = 0.004, ^##^*p* = 0.006. (F) Representative traces showing tonic inhibitory currents recorded from layer 5 pyramidal neurons in Sham + SH, Stroke + SH, and Stroke + EE mice. Blue arrows indicate baseline shift after blocking all GAT-1 receptors with NO-711 (10 µM). Black arrows indicate baseline shift after blocking all GABA_A_ receptors with BMI (100 µM). (G) Bar graph showing tonic current density after blocking GAT-1 receptors in the peri-infarct cortex. One-way ANOVA followed by post hoc Scheffe test, *F*_(2,38)_ = 4.92, ^*^p = 0.039, ^#^*p* = 0.032. (H) Bar graph showing the increased *I*_tonic_ produced by blocking GAT-1. One-way ANOVA followed by post hoc Scheffe test, *F*_(2,38)_ = 32.86, ^***^p < 0.001, ^###^*p* < 0.001. (I) Bar graph showing the amplitude of sIPSCs in the peri-infarct cortex after NO-711 (10 µM) application. One-way ANOVA followed by post hoc Scheffe test, *F*_(2,38)_ = 0.16, *p* = 0.8487. (J) Bar graph showing the frequency of sIPSCs in the peri-infarct cortex after NO-711 (10 µM) application. One-way ANOVA followed by post hoc Scheffe test, *F*_(2,38)_ = 0.02, *p* = 0.9787. BMI, bicuculline methiodide; GABA, gamma-aminobutyric acid; GAT, GABAtransporter; sIPSC, spontaneous inhibitory postsynaptic current.

GAT-1 preferentially expressed in neurons 32 limits tonic GABA currents and mediates phasic GABA inhibition 20, 21. Importantly, a recent bioinformatic study showed that EE exposure increased the density of the neurotransmitter gene co-expression network, in which *Gat-1* is a hub gene 22. We thus investigated whether EE modifies GAT-1. Given that EE reversed stroke-induced acetyl-H4 downregulation in the peri-infarct cortex (**Figure S2A-C**) and the expression and function of GAT-1 are critically regulated by BDNF 17, 33, we performed ChIP assays to investigate the effects of EE on GATs and BDNF genes, including *Gat-1*, *Gat-3*, and* Bdnf*. EE effectively reversed the stroke-induced downregulation of promoter region acetylation of *Gat-1*,* Bdnf-p1*, and* Bdnf-p4* but not *Gat-3* in the peri-infarct cortex (**Figure 3A**;** S3A**). More importantly, consistent with the ChIP assays, stroke-induced downregulation of GAT-1 expression in the peri-infarct cortex was rescued by EE exposure (**Figure 3B**). Furthermore, immunofluorescence of GAT-1 in the peri-infarct cortex also showed that EE relieved the decrease in GAT-1 caused by stroke (**Figure 3C**). To test whether EE inhibits GAT-1 internalization as BDNF does (17), membrane and cytosol protein fractions were extracted from the peri-infarct cortex and the corresponding cortex area of sham-operated mice. β-actin was detected in the cytosol fractions but not in the membrane protein fractions, while E-cadherin was detected in the membrane protein fractions but not in the cytosol protein fractions (**Figure S3B**), confirming the purity of the isolated membrane and cytosol proteins. Immunoblotting analysis showed that EE reversed the stroke-induced decrease in GAT-1 in the plasma membrane fraction and increase in the cytosol fraction (**Figure 3D-E**), suggesting that EE may modify the trafficking of GAT-1. To further explore the role of BDNF in GAT-1 trafficking, TrkB-Fc, a mature BDNF scavenger 34, was infused daily into the peri-infarct cortex of mice exposed to EE on days 5-11 after stroke. In accordance with the notion that BDNF inhibits GAT-1 internalization 17, TrkB-Fc had no effect on the total level of GAT-1, but decreased GAT-1 membrane fractions and increased GAT-1 cytosol fractions (**Figure S3C-E**), suggesting that BDNF deficiency leads to trafficking of GAT-1 from membrane to cytosol. Given that the beneficial effects of EE have been associated with chromatin remodeling 35, TSA, a pan HDAC inhibitor, was utilized *in vitro* to mimic EE conditioning *in vivo*. Immunofluorescence of GAT-1 in hESCs-derived neurons that underwent this mimetic ischemia confirmed that TSA reversed the GAT-1 internalization caused by stroke (**Figure S4A-D**). Consistent with the reported literature 17, 18, a small amount of GAT-1 was co-localized with astrocytes (**Figure S5A**). However, immunoblotting analysis showed that ischemia and TSA treatment had no effect on the expression of GAT-1 in astrocytes (**Figure S5B**), suggesting that EE exposure mainly upregulated the expression of GAT-1 in neurons. HDAC2 has been reported to be a critical factor in EE-mediated stroke 36. To test whether HDAC2 regulates the expression of GAT-1, LV-shHDAC1 or LV-shHDAC2 was microinjected into the motor cortex of mice 3 days before stroke to specifically knockdown peri-infarct HDAC1 or HDAC2, and immunoblotting was performed 5 days after stroke. Consistent with the notion that HDAC2 is a critical factor in EE-mediated stroke recovery 36, HDAC2 but not HDAC1 knockdown upregulated GAT-1 expression (**Figure S6A-D**). Thus, GAT-1 may be downstream of BDNF, although HDAC2 negatively regulates the expression of BDNF and GAT-1 at the same time. To understand the relationship between EE and GAT-1 function in regulating phasic and tonic GABA inhibition, we exposed mice to EE or SH on days 5-11 and performed electrophysiology on day 12 after stroke. We incubated acute brain slices from stroke mice with NO-711, a GAT-1-selective antagonist, and recorded tonic GABA currents and sIPSCs in the peri-infarct pyramidal neurons. NO-711-induced increases in *I*_tonic_ in sham and EE-treated stroke mice were significantly higher than those in SH-treated stroke mice (**Figure 3F-H**), suggesting that stroke caused GAT-1 dysfunction and EE restored GAT-1 function. Furthermore, NO-711 diminished sIPSC frequency in all groups (**Figure 3J** vs** Figure 1F**), suggesting that GAT-1 mediated phasic GABA inhibition through presynaptic mechanisms and NO-711 abolished the effect of EE on sIPSC frequency (**Figure 3I-J**), indicating that EE modifies phasic GABA inhibition through GAT-1. Together, these data demonstrate that EE positively regulates the expression and function of GAT-1 in the peri-infarct cortex during the repair phase of stroke.

### GAT-1 is necessary for EE-induced network plasticity after stroke

Chronically raised tonic inhibition antagonizes neuronal plasticity 5. To test whether GAT-1 dysfunction affects structural neuroplasticity, a basis for neuronal excitability, primary cultured cortical neurons were treated with OGD and then incubated with with TSA or a combination of TSA and NO-711. TSA rescued OGD-induced decreases in synapsin and postsynaptic density protein 95 (PSD-95)-positive puncta, an indicator of synaptogenesis, and NO-711 cancelled the effect of TSA (**Figure S7A-D**). The ratio between mushroom-like and thin spines (M/T) is an indicator of spine maturation 37. We found that TSA reversed OGD-induced M/T decline and NO-711 abolished the effect of TSA (**Figure S7E-F**). Thus, GAT-1 function is implicated in the epigenetic regulation of structural neuroplasticity.

**Figure 4:**
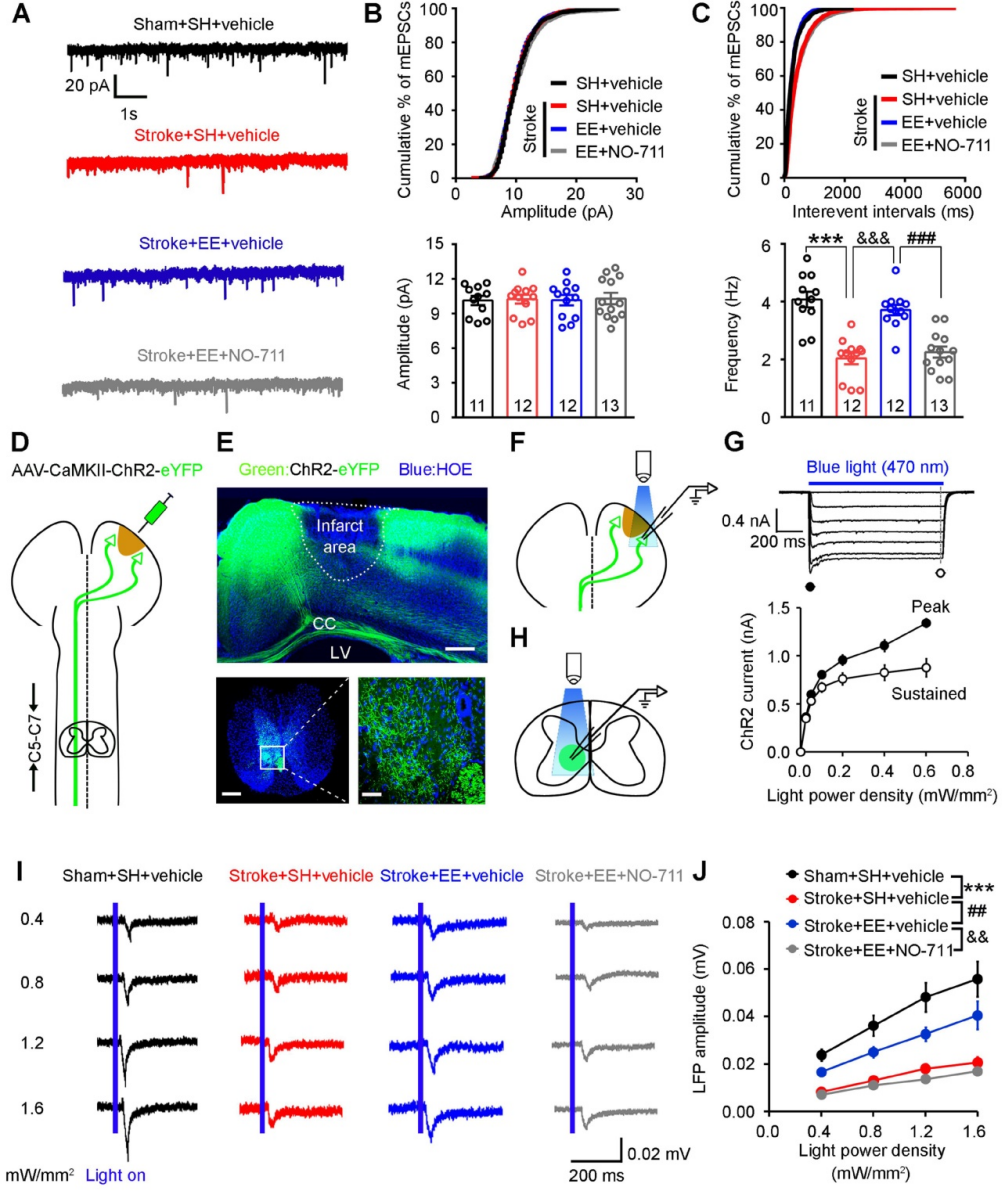
** Requirement of GAT-1 for EE-induced input-output synaptic strength enhancement after stroke.** (A-C) Representative mEPSC traces (A), and mEPSC amplitude (B) and frequency (C) recorded in peri-infarct pyramidal neurons from mice of the indicated groups. One-way ANOVA followed by post hoc Scheffe test, *F*_(3,44)_ = 22.96, ^***^p < 0.001, ^&&&^*p* < 0.001, ^###^*p* < 0.001. (D) Schematic diagram showing injection of AAV-CaMKII-ChR2 into the peri-infarct cortex to label axons descending in the corticospinal tract. (E) Top, representative images showing peri-infarct cortex infected with AAV-CaMKII-ChR2. Scale bar, 500 µm. Bottom representative image of mouse corticospinal tract axons projecting from the peri-infarct cortex (scale bar, 500 µm) with a magnified view of the selected area shown on the right (scale bar, 50 µm). (F) Schematic diagram showing patch-clamp recording of a peri-infarct ChR2-GFP-positive pyramidal neuron and optogenetic stimulation of the neuron. (G) Top, ChR2-mediated currents in peri-infarct pyramidal neurons evoked by 1 s pulses of light with variable intensities (0.0-0.6 mW/mm^2^) at -70 mV in voltage-clamp mode in the presence of NBQX (10 µM) and BMI (20 µM). Bottom, input-output curves of ChR2-mediated photocurrents showing peak (filled circles) and sustained (open circles) current components plotted as a function of light intensity (n = 10 neurons from 3 animals). (H) Schematic diagram showing LFPs in the C5-C7 cervical spinal cord segment recorded by optogenetic stimulation of ChR2-GFP-positive axons projecting from peri-infarct pyramidal neurons. (I) Representative LFP traces (average of 20 responses) in the corticospinal tract in mice of the indicated groups (n = 13 slices from 5-6 animals for each group). (J) Input-output curves of peak amplitude for LFPs from the peri-infarct cortex to the spinal cord pathway. Two-way repeated-measures ANOVA followed by post hoc Bonferroni test, *F*_(3,48)_ = 22.49, ^***^p < 0.001, ^##^*p* = 0.008, ^&&^*p* = 0.001. BMI, bicuculline methiodide; CC, corpus callosum; LFP, local field potential; LV, lateral ventricle; mEPSC, miniature excitatory post-synaptic current.

Dendritic spines are the postsynaptic sites of excitatory synapses and receive excitatory synaptic inputs 38. To determine whether GAT-1 is required for EE-mediated increases in excitatory synaptic transmission after ischemia, we exposed mice to EE or SH and infused NO-711 or vehicle daily into the peri-infarct cortex of conscious mice via an implanted microcannula on days 5-11 after stroke. As shown in (**Figure 4A-C**), mEPSC frequency was significantly decreased in SH-treated mice subjected to stroke compared to sham-operated mice, exposure to EE reversed the stroke-induced decrease in mEPSC frequency, and NO-711 abolished the promotion effect of EE on mEPSCs, indicating that GAT-1 is critical for EE-induced input synaptic strengthening in the peri-infarct cortex.

Corticospinal tract (CST) lesion is a biomarker for stroke motor outcomes 36, 39. EE has been shown to promote axon regeneration in rodent spinal cord injury models 40 and improve CST plasticity after traumatic brain injury 41. To evaluate whether GAT-1 is implicated in the EE-mediated increase in the plasticity of CST after stroke, we transduced peri-infarct pyramidal neurons with an AAV encoding ChR2-eYFP fusion gene under control of the CaMKII promoter (**Figure 4D**). Three weeks later, ChR2-eYFP, identified as green fluorescence, was abundantly expressed in surviving neurons in the peri-infarct cortex (**Figure 4E top**). Furthermore, ChR2-expressing peri-infarct motor cortex fibers abundantly projected to the C5-C7 cervical spinal cord segment (**Figure 4E bottom**). To validate the functionality of expressed ChR2, we illuminated the peri-infarct cortex in the acute slices with blue light (470 nm) (**Figure 4F**). The illumination resulted in inward currents in transduced pyramidal neurons under voltage-clamp recording conditions at a holding potential of -70 mV that followed the light power density, confirming the functionality of expressed ChR2 (**Figure 4G**). Next, we recorded LFPs in the C5-C7 cervical spinal cord segment by photostimulating the fibers projected from the peri-infarct cortex (**Figure 4H**). Stroke caused a substantial decrease in synaptic responses in the CST, which was ameliorated by EE exposure. Interestingly, NO-711 abolished the EE-dependent enhancement in LFPs (**Figure 4I-J**). Thus, GAT-1 is required for EE-induced output synaptic strengthening in the CST.

**Figure 5:**
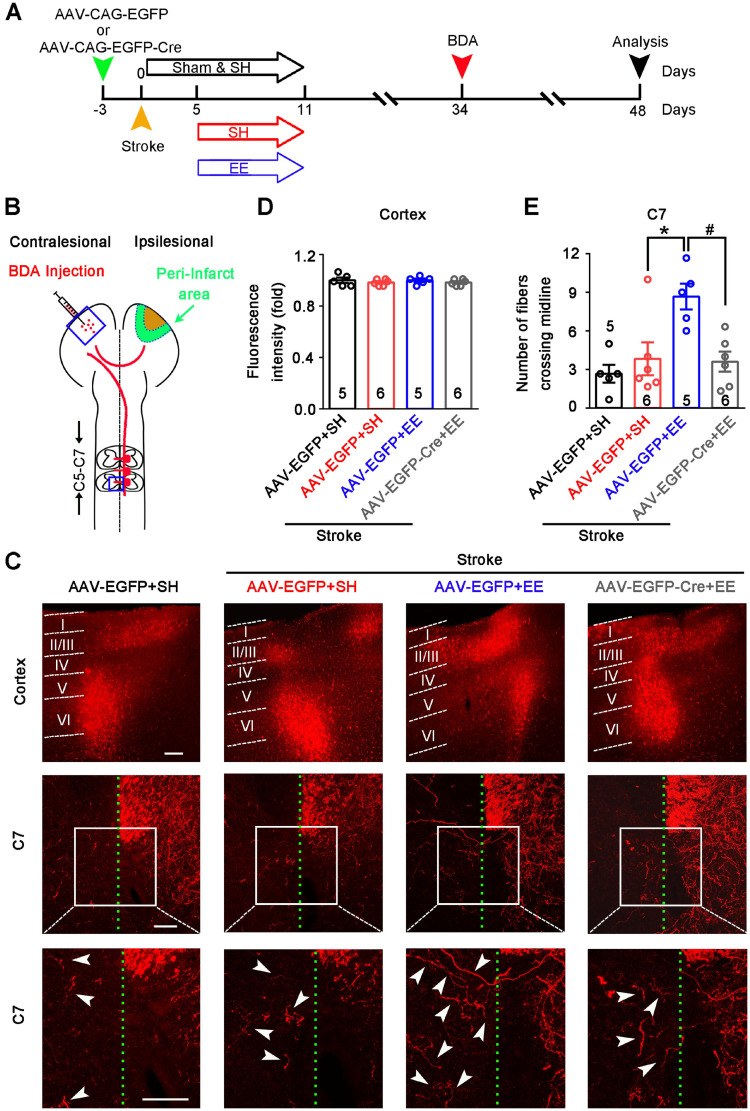
** GAT-1 is critical for EE-induced sprouting of uninjured corticospinal axons after stroke.** (A) Experimental design for (B-E). AAV-CAG-EGFP or AAV-CAG-EGFP-Cre was microinjected into the motor cortex of GAT-1*^flox/flox^*mice 3 days before stroke. Mice were exposed to SH or EE on days 5-11 after stroke. BDA was injected into the contralesional motor cortex on day 34 after stroke. Then, the brains and spinal cords were harvested for BDA fluorescence signal examination 14 days after BDA injection. (B) Schematic diagram of tract tracing experiments. (C) Representative images showing BDA^+^ labelling in the contralesional motor cortex (top) and, the C7 spinal cord (middle). High-magnification images from selected areas in the middle images are shown on the bottom. The midline is depicted in green, and white arrows indicate sprouting axons at the level of C7. Scale bar, 100 µm. (D) Quantification of BDA fluorescence intensity in the cortex (3 images per animal). One-way ANOVA followed by post hoc Scheffe test, *F*_(3,18)_ = 0.49. (E) Quantification of the number of midline-crossing axons per section in the C7 spinal cord (3 images per animal). One-way ANOVA followed by post hoc Scheffe test, *F*_(3,18)_ = 6.84, ^*^p = 0.025, ^#^*p* = 0.019. BDA, biotinylated dextran amine; EE, environmental enrichment; SH, standard housing.

Axonal sprouting is an attractive mechanism underlying the restoration of neuronal networks and functions after stroke 25. Sprouting of uninjured corticospinal axons across the midline into the territory of denervated spinal cord reflects the plasticity of the CST, which contributes to motor functional recovery after stroke and traumatic brain injury 41-43. To determine whether GAT-1-mediated neuronal plasticity in the peri-infarct cortex is necessary for EE-induced sprouting of uninjured corticospinal axons, we generated a GAT-1*^flox/flox^*mouse line (**Figure S8**). We infused AAV-CAG-EGFP-Cre into the motor cortex of GAT-1*^flox/flox^*mice to specifically delete local GAT-1. Stroke was induced 3 days after virus microinjection. Then, mice were exposed to EE or SH on days 5-11 after stroke. The tract tracer BDA was microinjected into the contralesional cortex on day 34 after stroke (**Figure 5A-B**). Fourteen days later, we performed fluorescence staining of BDA in the contralesional motor cortex and spinal cord. BDA fluorescence in the contralesional motor cortex was comparable between groups, suggesting that BDA injection was consistent (**Figure 5C-D**). EE exposure significantly increased the number of BDA^+^ fibers crossing the midline into the denervated spinal cord at the C7 level, and the EE-induced sprouting of uninjured corticospinal axons was abolished by deleting GAT-1 in the peri-infarct cortex (**Figure 5C, E**). Together, the function of GAT-1 in the peri-infarct cortex is crucial for EE-induced neuronal plasticity in the cortex and CST after stroke.

### GAT-1 is critical for functional recovery from stroke

**Figure 6:**
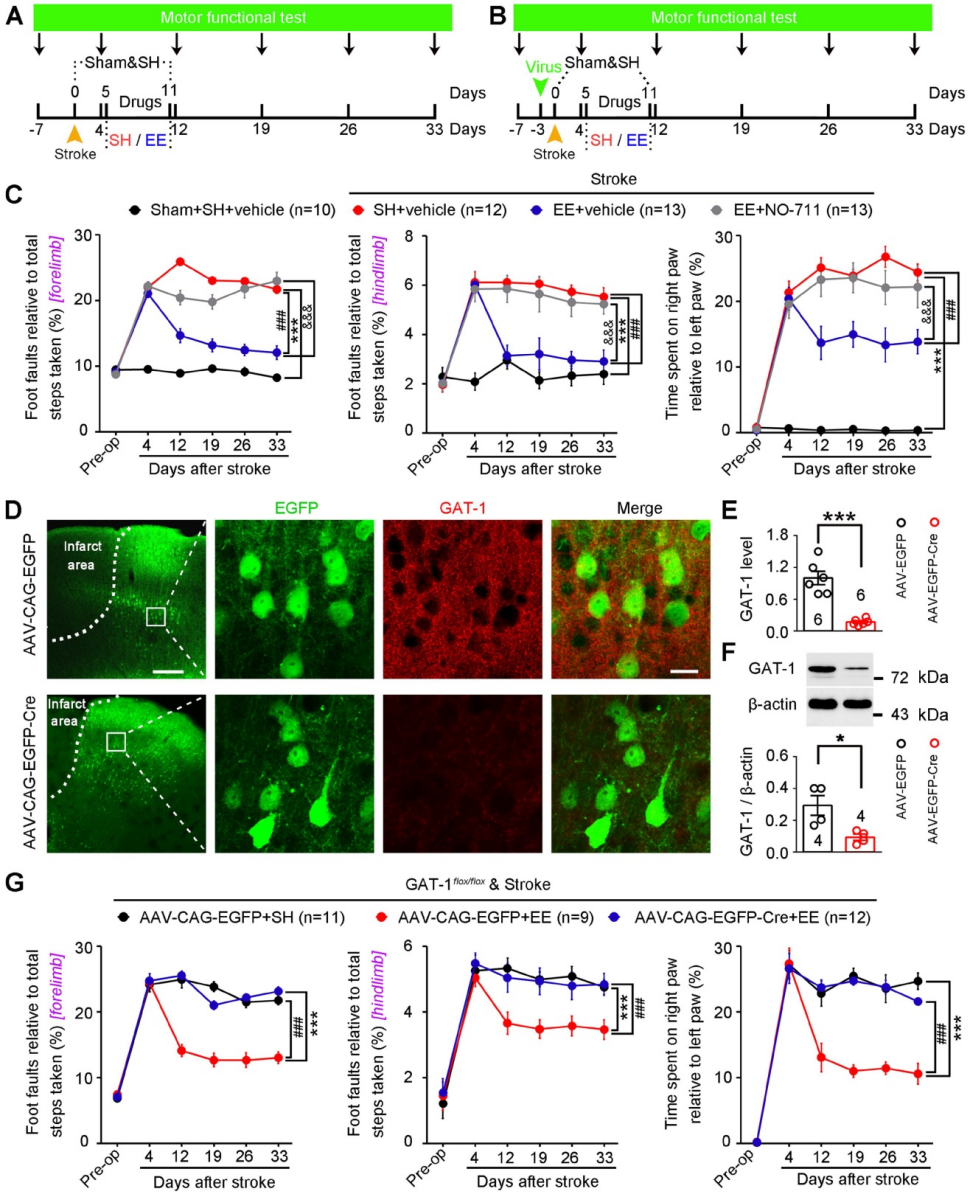
** GAT-1 is necessary for EE-mediated functional recovery after stroke.** (A) Experimental design for (C). NO-711 (10 µM in 2 µL each day) was microinjected into the peri-infarct cortex on days 5-11 after stroke, and motor function was measured 7 days before and 4, 12, 19, 26, and 33 days after stroke. (B) Experimental design for (D-G). AAV-CAG-EGFP or AAV-CAG-EGFP-Cre was microinjected into the motor cortex of GAT-1*^flox/flox^*mice 3 days before stroke, and motor function was measured 7 days before and 4, 12, 19, 26, and 33 days after stroke. (C) Left, foot faults of the left forelimb in the grid-walking task. Middle, foot faults of the left hindlimb in the grid-walking task. Right, forelimb symmetry in the cylinder task. Two-way repeated-measures ANOVA followed by post hoc Bonferroni test. Left, *F*_(3,44)_ = 225.755, ^***^p < 0.001, ^###^*p* < 0.001, ^&&&^*p* < 0.001. Middle, *F*_(3,44)_ = 56.852, ^***^p < 0.001, ^###^*p* < 0.001, ^&&&^*p* < 0.001. Right, *F*_(3,44)_ = 117.328, ^***^p < 0.001, ^###^*p* < 0.001, ^&&&^*p* < 0.001. (D) Representative images showing AAV-infected peri-infarct cortex 5 days after stroke and high-magnification images from selected areas that were stained for EGFP and GAT-1. Left scale bar, 100 µm. Right scale bar, 20 µm. (E) Bar graph showing GAT-1 fluorescence intensity in the peri-infarct cortex 8 days after AAV-CAG-EGFP or AAV-CAG-EGFP-Cre infection. Two-tailed *t*-test, *F*_(1,10)_= 41.8, ^***^p < 0.001. (F) GAT-1 level in the peri-infarct cortex 8 days after AAV-CAG-EGFP or AAV-CAG-EGFP-Cre infection. Two-tailed *t*-test, *F*_(1,6)_= 9.3, ^*^p = 0.0225. (G) Left, foot faults of the left forelimb in the grid-walking task. Middle, foot faults of the left hindlimb in the grid-walking task. Right, forelimb symmetry in the cylinder task. Two-way repeated-measures ANOVA followed by post hoc Bonferroni test. Left, *F*_(2,29)_ = 119.657, ^***^p < 0.001, ^###^*p* < 0.001. Middle, *F*_(2,29)_ = 17.930, ^***^p < 0.001, ^###^*p* < 0.001. Right, *F*_(2,29)_ = 45.919, ^***^p < 0.001, ^###^*p* < 0.001. EE, environmental enrichment; SH, standard housing.

Owing to the difficulties in translating EE from the bench to the bedside in stroke 13, 16, identifying key molecular substrates of the effects of EE on stroke recovery is of great significance for developing effective pharmacological agents. To identify possible targets, we induced focal cerebral ischemia by precise photothrombosis of mouse motor cortex, which resulted in long-lasting motor cortex injury (**Figure S9**). To determine whether GAT-1 can serve as the target molecule, mice were exposed to EE or SH and NO-711 or vehicle was infused daily into the peri-infarct cortex on days 5-11 after stroke. Motor function was measured 7 days before stroke and 4, 12, 19, 26, and 33 days after stroke (**Figure 6A**). Mice subjected to photothrombotic stroke in the forelimb motor cortex showed a marked impairment in motor function compared to sham-operated mice. The mice exposed to EE displayed significantly better motor function than mice exposed to SH on days 12-33 after stroke, and treatment with NO-711 abolished the EE-mediated functional recovery from stroke (**Figure 6C**). To further confirm whether GAT-1 is a key mediator for EE-dependent functional recovery, AAV-CAG-EGFP-Cre was microinjected into the motor cortex of GAT-1*^flox/flox^*mice 3 days before stroke to specifically delete peri-infarct GAT-1, and motor function was measured 7 days before and 4, 12, 19, 26, and 33 days after stroke (**Figure 6B**). GAT-1 was almost completely deleted in the AAV-Cre-infected cells in the peri-infarct cortex (**Figure 6D-F**). In wild-type animals, EE exposure significantly ameliorated motor function, while it had no effect in mice with GAT-1 conditional knockout in the peri-infarct cortex (**Figure 6G**), suggesting that GAT-1 is essential for EE-induced functional recovery after stroke.

**Figure 7:**
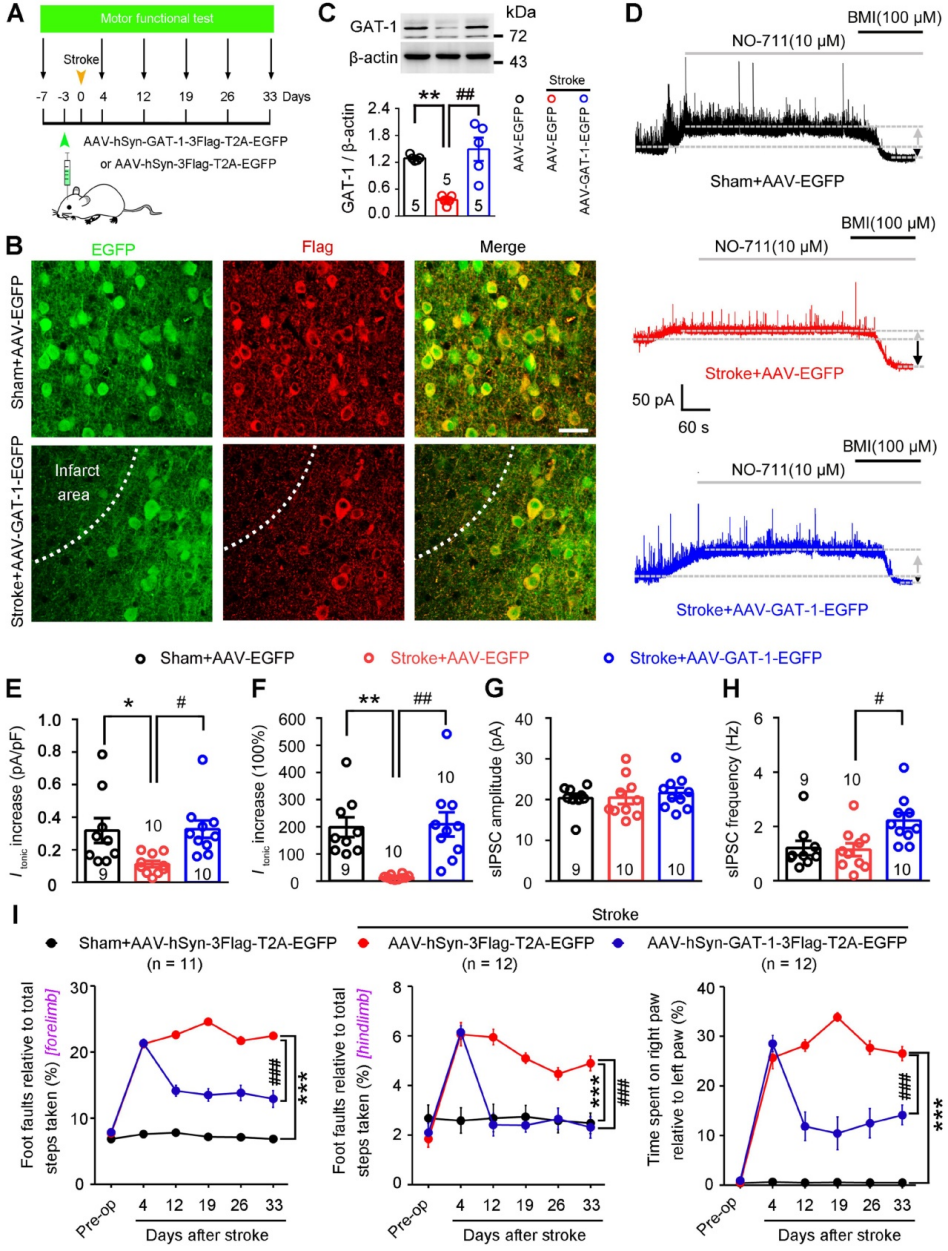
** GAT-1 overexpression promotes functional recovery after stroke.** (A) Experimental design for (B-I). (B) Representative images showing AAV-infected sham-operated and peri-infarct cortex 5 days after stroke and high-magnification images from selected areas that were stained for EGFP and Flag. Scale bar, 40 μm. (C) Immunoblots and bar graph showing GAT-1 level in the peri-infarct cortex 8 days after AAV-EGFP or AAV-GAT-1-EGFP infection. One-way ANOVA followed by post hoc Scheffe test, *F*_(2,12)_ = 15.39, ^**^p = 0.004, ^##^*p* = 0.001. (D) Representative traces showing tonic inhibitory currents recorded from layer 5 pyramidal neurons in Sham + AAV-EGFP, Stroke + AAV-EGFP, and Stroke + AAV-GAT-1-EGFP mice. Grey arrows indicate baseline shift after blocking all GAT-1 receptors with NO-711 (10 μM). Black arrows indicate baseline shift after blocking all GABA_A_ receptors with BMI (100 μM). (E) Bar graph showing tonic current density after blocking GAT-1 in the peri-infarct cortex. One-way ANOVA followed by post hoc Scheffe test, *F*_(2,26)_ = 5.37, ^*^p = 0.038, ^#^*p* = 0.026. (F) Bar graph showing the increased *I*_tonic_ produced by blocking GAT-1. One-way ANOVA followed by post hoc Scheffe test, *F*_(2,26)_ = 11.03, ^**^p = 0.003, ^##^*p* = 0.001. (G) Bar graph showing the amplitude of sIPSCs in the peri-infarct cortex 8 days after AAV-hSyn-3Flag-T2A-EGFP or AAV-hSyn-GAT-1-3Flag-T2A-EGFP infection. One-way ANOVA followed by post hoc Scheffe test, *F*_ (2,26)_ = 0.27. (H) Bar graph showing the frequency of sIPSCs in the peri-infarct cortex 8 days after AAV-hSyn-3Flag-T2A-EGFP or AAV-hSyn-GAT-1-3Flag-T2A-EGFP infection. One-way ANOVA followed by post hoc Scheffe test, *F*_ (2,26)_ = 5.44, ^#^*p* = 0.023. (I) Left, foot faults of the left forelimb in the grid-walking task. Middle, foot faults of the left hindlimb in the grid-walking task. Right, forelimb symmetry in the cylinder task. Two-way repeated-measures ANOVA followed by post hoc Bonferroni test. Left, *F*_(2,32)_ = 758.252, ^***^p < 0.001, ^###^*p* < 0.001. Middle, *F*_(2,32)_ = 44.922, ^***^p < 0.001, ^###^*p* < 0.001. Right, *F*_(2,29)_ = 311.493, ^***^p < 0.001, ^###^*p* < 0.001. AAV, adeno-associated virus; BMI, bicuculline methiodide; GABA, gamma-aminobutyric acid; sIPSC, spontaneous inhibitory postsynaptic current.

To directly evaluate the role of GAT-1 in functional recovery from stroke, we generated an AAV vector expressing GAT-1 (AAV-hSyn-GAT-1-3Flag-T2A-EGFP) and its control AAV-hSyn-3Flag-T2A-EGFP. We infused these vectors into the motor cortex of mice 3 days before stroke, performed immunoblots, immunofluorescence staining, and electrophysiological recordings on day 5 after stroke, and measured motor function 7 days before and 4, 12, 19, 26, and 33 days after stroke (**Figure 7A**). AAV-GAT-1-3Flag effectively infected the peri-infarct cortex (**Figure 7B**) and significantly upregulated GAT-1 in the peri-infarct cortex (**Figure 7C**). Furthermore, as an important control of GAT-1 overexpression, we generated an AAV vector expressing GAT-3 (AAV-CAG-GAT-3-3Flag-T2A-mCherry) and its control AAV-CAG-3Flag-T2A-mCherry. We infused these vectors into the motor cortex of mice 3 days before stroke, and performed immunoblots on day 5 after stroke. The immunoblots confirmed that AAV-CAG-GAT-3-3Flag-T2A-mCherry significantly upregulated GAT-3 in the peri-infarct area (**Figure S10A**). To visualize peri-infarct pyramidal apical dendritic spines, LV-Ubi-GFP was microinjected into the motor cortex of mice 10 days before stroke. AAV-hSyn-GAT-1-3Flag or AAV-CAG-GAT-3-3Flag-T2A-mCherry was microinjected 7 days later. Immunofluorescence staining was performed 5 days after stroke. GAT-1 but not GAT-3 overexpression attenuated the stroke-induced decrease in spine density in the peri-infarct area (**Figure S10B-C**), suggesting that GAT-1 is critical for structural plasticity after stroke. The NO-711-induced increase in* I*_tonic_ in AAV-control-treated sham mice was significantly higher than that in AAV-control-treated stroke mice (**Figure 7D-F**), again confirming GAT-1 dysfunction in peri-infarct neurons. A similar trend was observed in mice treated with AAV-GAT-1-3Flag (**Figure 7D-F**), suggesting restoration of GAT-1 function. Moreover, peri-infarct pyramidal neurons from AAV-GAT-1-3Flag-treated mice showed significantly increased sIPSC frequency but not amplitude compared to AAV-control-treated sham and stroke mice (**Figure 7G-H**). To test whether GAT-3 overexpression can rescue electrophysiological defects in GAT-1 conditional knockout neurons, AAV-CAG-EGFP-Cre was microinjected into the motor cortex of GAT-1*^flox/flox^*mice 10 days before stroke to specifically delete peri-infarct GAT-1. AAV-CAG-GAT-3-3Flag-T2A-mCherry was microinjected into the motor cortex of GAT-1*^flox/flox^*mice 7 days later to overexpress GAT-3. Tonic inhibitory currents were recorded 5 days after stroke. Importantly, GAT-3 overexpression did not rescue the GAT-1 knockdown-induced increase in *I*_tonic_ in peri-infarct layer 5 pyramidal neurons (**Figure S10D-E**). Collectively, overexpression of GAT-1 restored the roles of GAT-1 in regulating phasic and tonic inhibition in the peri-infarct cortex. Importantly, overexpression of GAT-1 but not GAT-3 significantly ameliorated stroke-induced functional impairment 12 to 33 days after stroke compared with AAV-control-treated stroke mice (**Figure 7I**;** S10F**). Thus, GAT-1 function is crucial for functional recovery from stroke.

## Discussion

With advances in stroke treatments for the acute time window, more patients survive stroke but have varying degrees of disability 1, 3. The brain and spinal cord undergo adaptive plasticity following stroke 6, 7, 44. EE shows great promise in promoting functional recovery from stroke by enhancing adaptive plasticity 12, 13, 44. However, EE remains a laboratory phenomenon with little clinical translation due to the complexities of utilizing EE in a clinical setting 13, 16. Enriched cages are equipped with running wheels and colored toys, which enhance motor and visual stimuli, respectively 23. Moreover, opportunities for social interactions increased compared with SH as 6 mice live together and interact 23. Although the beneficial effect of EE on stroke recovery is a synergistic effect with the enhanced neuroplasticity of the whole brain, neuroplasticity in the peri-infarct area is critical for functional recovery after stroke 5, 13. We show here that EE exposure upregulates the expression of GAT-1 and inhibits internalization of GAT-1 from the membrane surface, which is a working state of GAT-1. Importantly, upregulation of GAT-1 in the peri-infarct cortex was necessary for EE-induced network plasticity in the ipsilesional and contralesional motor cortex and CST, and for motor function recovery after stroke. Moreover, overexpression of GAT-1 in the peri-infarct cortex ameliorated stroke-induced functional impairment, similar to EE. Thus, GAT-1 is a key mediator of EE in remapping motor and sensory function after stroke and can serve as a novel therapeutic target.

Extracellular concentration of GABA is effectively controlled by GATs, which critically determines tonic conductance, neuronal excitability threshold, and LTP induction 5. GABA acting at GABAARs mediates tonic and phasic inhibition, with the former regulating membrane potential and network excitability 21 and the latter regulating point-to-point inter-neuronal communication, preventing the overexcitation of neurons and thereby avoiding the development of pathological states of network activity 45. While tonic inhibition appears to suppress brain repair 5, enhancing phasic GABA signaling improves functional recovery from stroke 19. GAT-1 in neurons regulates GABA levels at the synaptic cleft and is responsible for phasic inhibition, while GAT-1 and GAT-2/3 in astrocytes work together extrasynaptically to limit tonic currents 20, 21. Although extracellular GABA is taken up by GATs under physiological conditions, GABA release through reversed GATs in reactive astrocytes can occur under some pathological conditions, including Alzheimer's disease 46 and stroke 27. The dual nature of glial GATs makes them unsuitable as pharmacological targets. Fortunately, GAT-1 reversal does not occur during physiological or pathological network activity 18. Moreover, stroke and EE changed GAT-1 but not GAT-3 (**Figure 3**). Therefore, GAT-1 but not other GATs, is a good target for treating stroke during the repair phase.

Structural and functional plasticity is fundamental for functional recovery from stroke 4, 47, 48. Excitability enhancement of neuronal networks facilitates plasticity and thereby promotes stroke recovery 8-10. Enhancing excitability by decreasing tonic GABA inhibition during the repair phase of stroke has been found to significantly improve functional recovery 5. We found that EE-induced stroke recovery depended on cortex excitability (**Figure 2A-E**). Although tonic GABAergic inhibition controls network excitability 5, a recent multicenter, double-blind, randomized, and placebo-controlled trial showed that S44819, a selective α5-containing GABA_A_R antagonist that reverses tonic inhibition in peri-infarct tissue, did not improve clinical outcome in patients after stroke 49. The α5-containing GABA_A_R exhibits both synaptic phasic and extrasynaptic tonic inhibition 50. While α5 GABA_A_R antagonist enhances network excitability by reducing tonic inhibition, blocking α5 GABA_A_R may also impair GABAergic synaptic transmission, a process that contributes to the homeostasis of circuit excitability 51. Possible risks of indiscriminately reducing both GABA-mediated phasic and tonic inhibition need to be determined because of the incidence of seizure after stroke 52 and the beneficial effects of phasic GABA signaling enhancement on stroke recovery 19. We found that EE exposure reduced tonic but increased phasic inhibition (**Figure 1C-F**). Additionally, elimination of phasic GABA signaling by silencing the activity of GABAergic inhibitory neurons abolished the effect of EE on functional recovery from stroke (**Figure 2A, F-I**). Therefore, reducing tonic and enhancing phasic GABA inhibition at the same time may modify network excitability in a regular and controlled manner, thereby improving stroke recovery. Indeed, regular plasticity is of critical importance for stroke recovery 43. Targeting GAT-1 may be a better therapeutic strategy for stroke than other approaches that only reduce tonic inhibition or increase phasic inhibition, as GAT-1 mediates phasic inhibition while reducing tonic inhibition 53.

BDNF is crucial for EE-induced neuroplasticity and stroke recovery 13, 23. However, clinical use of BDNF is limited by its difficulty in penetrating the blood brain barrier and short tissue distribution time. Although TrkB agonists also promote recovery from stroke, systemic BDNF/TrkB signaling throughout the body may cause off-target effects outside of the brain 54. HDAC-mediated histone acetylation is also involved in EE-induced neuroplasticity 35, 55. HDAC inhibitor effectively reverses stroke-induced downregulation of promoter region acetylation of *Bdnf* in the peri-infarct cortex and promotes stroke recovery 26. However, HDAC inhibitors are often toxic or have unwanted side effects in humans 56-58. Similar to HDAC inhibitors, we found that EE reverses stroke-induced downregulation of promoter region acetylation of *Bdnf* (**Figure S3A**). Simultaneously, the expression and membrane localization of GAT-1 are positively regulated by BDNF (**Figure S3C-E**) and associated with the beneficial effects of EE 59. Moreover, EE-induced network plasticity and stroke recovery depend on GAT-1 function (**Figure 4-6**). Thus, targeting GAT-1 may have advantages over other EE-related target molecules, such as BDNF and HDACs.

## Conclusions

**Figure 8:**
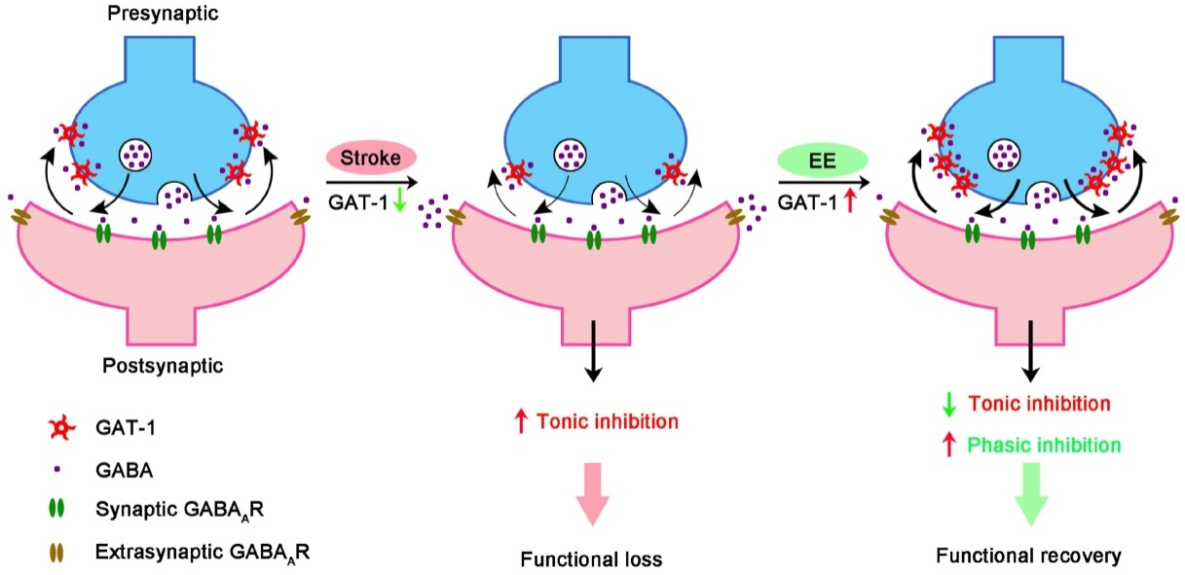
GAT-1 dysfunction in the peri-infarct cortex enhances tonic inhibition, thereby impairing stroke recovery. EE reduces tonic inhibition while facilitating phasic inhibition by restoring GAT-1 function and consequently promotes stroke recovery.

In summary, our findings indicate that EE-induced network plasticity enhancement and consequent stroke recovery during the repair phase depend on GAT-1-mediated amplification of phasic GABA signaling and reduction of tonic GABA signaling (**Figure 8**). From these results, we identify a novel therapeutic target for functional recovery from stroke. Drugs targeting GAT-1 may mimic the impact of EE and offer a novel therapeutic strategy for stroke recovery within a feasible time window. Given that EE-induced network plasticity benefits various neuropsychiatric disorders, targeting GAT-1 may bring hope to the treatment of other diseases and is not limited to stroke.

## Supplementary Material

Supplementary figures.

## Abbreviations

AAV: adeno-associated virus
ACSF: artificial cerebrospinal fluid
AP: action potential
BDA: biotinylated dextran amine
BDNF: brain-derived neurotrophicfactor
BMI: bicuculline methiodide
CC: corpus callosum
ChIP: chromatin immunoprecipitation
ChR2: channelrhodopsin-2
CNO: clozapine-*N*-oxide
CNS: central nervous system
CST: corticospinal tract
DIV: days *in vitro*
EE: environmental enrichment
GAT: GABA transporter
HDAC: histone deacetylase
hESC: human embryonic stem cell
LED: light-emitting diode
LFP: local field potential
LV: lateral ventricle
mEPSC: miniature excitatory postsynaptic current
OGD: oxygen glucose deprivation
PCR: polymerase chain reaction
PFA: paraformaldehyde
RRID: research resource identifier
SEM: standard error of the mean
SH: standard housing
sIPSC: spontaneous inhibitory post-synaptic current
sgRNA: single-guide RNA
TSA: trichostatin A
