# Supplementary material for: Environmental enrichment implies GAT-1 as a potential therapeutic target for stroke recovery
Source: Theranostics. 2021 Jan 27;11(8):3760–80. doi: 10.7150/thno.53316 (PMC7914370; doi:10.7150/thno.53316)
Supplement: Supplementary file 1 — Supplementary figures. [file thnov11p3760s1.pdf]

Supplementary Figures

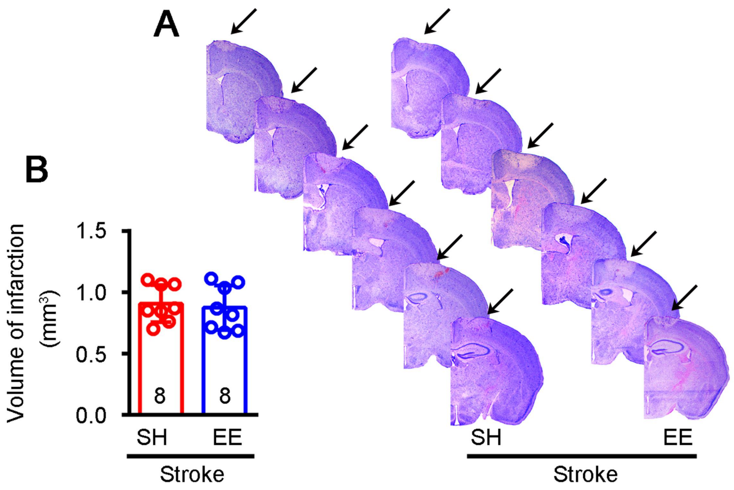

Figure S1. EE exposure 5–11 days after stroke has no effect on infarct size. **(A)** Representative Nissl-stained sections 12 days after stroke from Stroke + SH and Stroke + EE mice. **(B)** Bar graph showing stroke volume in Stroke + SH and Stroke + EE mice. Two tailed *t*-test, $F_{(1,14)} = 0.16$. EE, environmental enrichment; SH, standard housing.

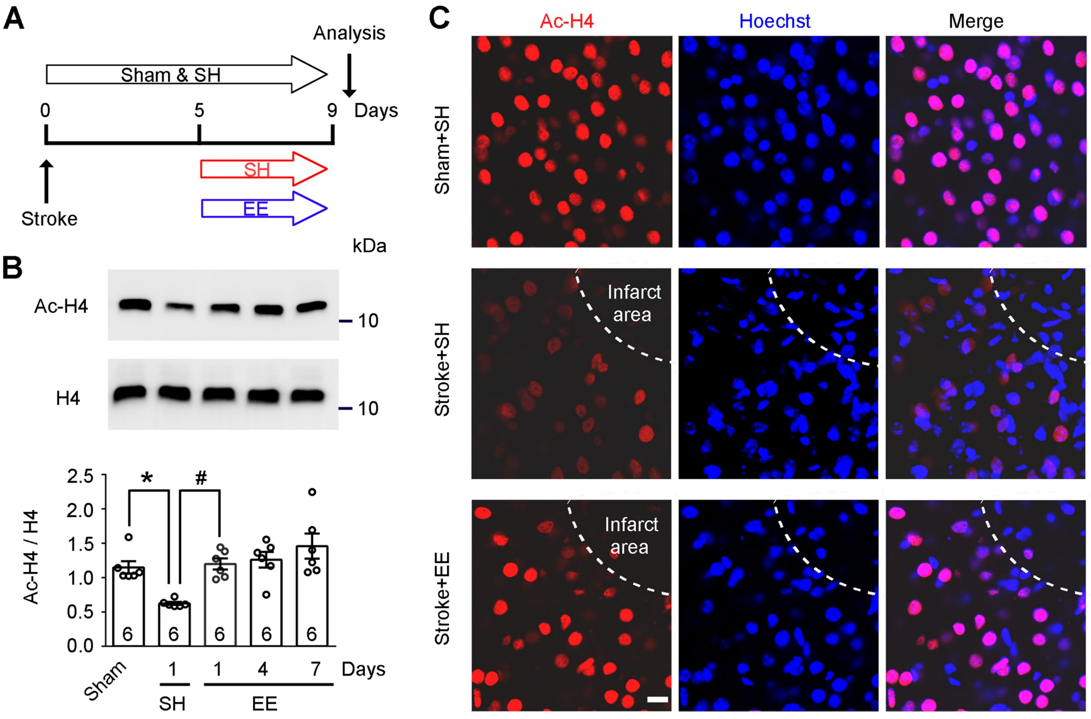

Figure S2. EE exposure induces chromatin modifications in the peri-infarct area. **(A)** Experimental design for **(C)**. **(B)** Representative immunoblots and bar graph showing the time course of acetyl-H4 levels in the peri-infarct cortex. Mice were exposed to SH or EE 5 days after stroke. One-way ANOVA followed by post hoc Scheffe test, $F_{(4,25)} = 7.81$, ${}^{*}p = 0.048$, ${}^{\#}p = 0.025$. **(C)** Representative immunofluorescence images showing acetyl-H4 in the peri-infarct cortex. Scale bar, 20 μm. EE, environmental enrichment; SH, standard housing.

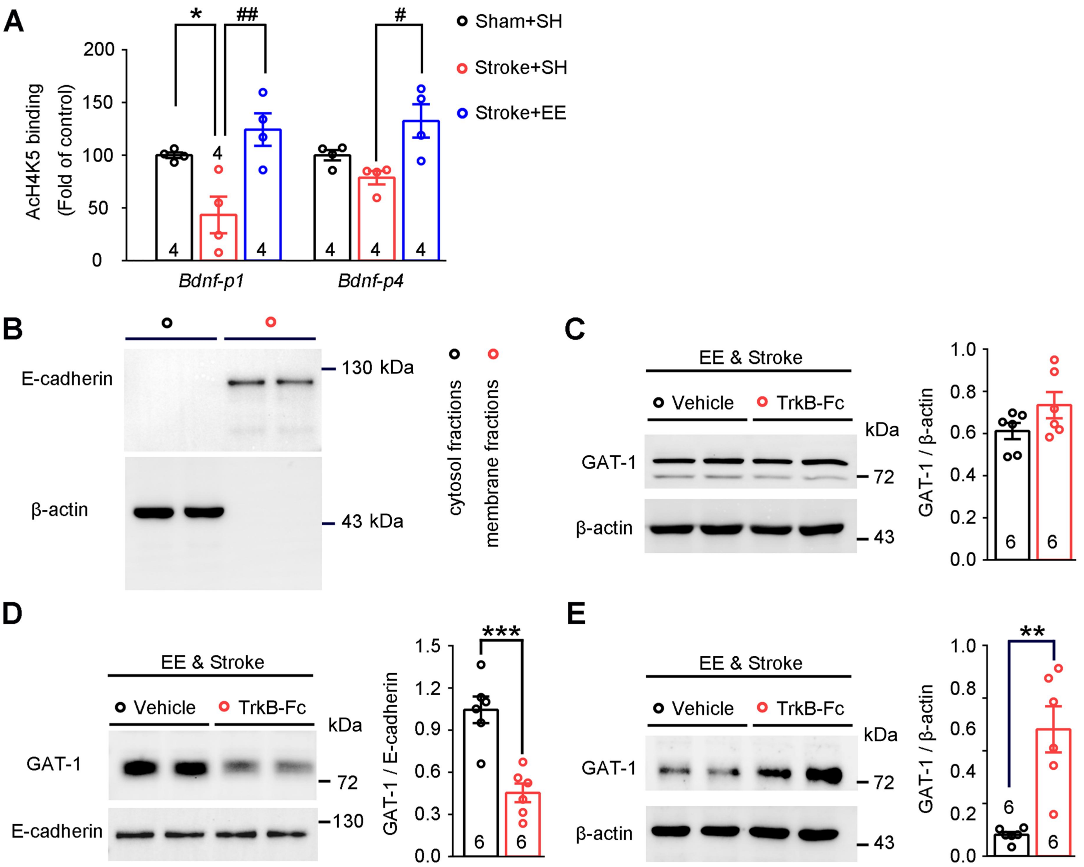

Figure S3. EE exposure primes a transcriptional program for the expression of BDNF, which regulates trafficking of GAT-1.

(A) Bar graph showing changes in histone acetylation in the promoter regions of BDNF genes in peri-infarct tissue. Fragmented chromatin was immunoprecipitated with antibody recognizing acetyl-H4 and quantified with real-time polymerase chain reaction. One-way ANOVA followed by post hoc Scheffe test. For *Bdnf-p1*, $F_{(2,9)}$ = 9.44, $^{*}p$ = 0.047, $^{\#\#}p$ = 0.007; for *Bdnf-p4*, $F_{(2,9)}$ = 7.01, $^{\#}p$ = 0.015. **(B)** Confirmation of the membrane protein fractions extraction method. Representative immunoblots showing that β-actin was exclusively detected in cytosol fractions, while E-cadherin was exclusively detected in membrane fractions. **(C)** Representative immunoblots and

bar graph showing GAT-1 content in peri-infarct cortex. Two-tailed *t*-test, $F_{(1,10)}$ = 2.82. **(D)** Representative immunoblots and bar graph showing GAT-1 content in membrane fraction. Two-tailed *t*-test, $F_{(1,10)}$ = 25.96, $^{***}p < 0.001$. **(E)** Representative immunoblots and bar graph showing GAT-1 content in cytosol fractions. Two-tailed *t*-test, $F_{(1,10)}$ = 20.58, $^{**}p = 0.001$. BDNF, brain-derived neurotrophicfactor.

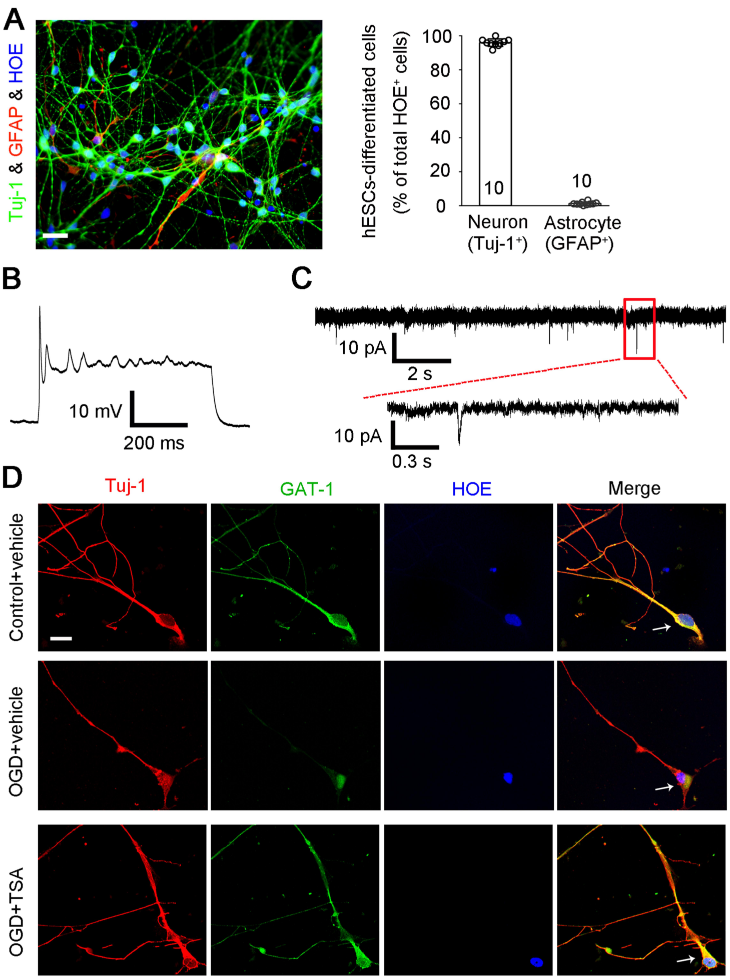

Figure S4. TSA reverses OGD-induced internalization of GAT-1 in hESCs-derived neurons.

(A) Representative fluorescence image and bar graph showing the percentage of neurons and astrocytes in hESCs-derived cells. Scale bar, 40 μm. **(B)** Representative whole-cell patch clamp trace illustrating the number of APs in hESCs-derived neurons evoked by current injection. **(C)** Representative voltage clamp (−70 mV) traces showing hESCs-derived neurons displaying spontaneous postsynaptic currents. **(D)** hESCs-derived neurons were exposed to 3 h of OGD. TSA (0.5 μM) was applied for 24 h, starting 24 h after OGD. Representative fluorescence images showing Tuj-1 and

GAT-1 in hESCs-derived neurons at 48 h after OGD. Scale bar, 20 μm. AP, action potential; hESC, human embryonic stem cell; OGD, oxygen glucose deprivation; TSA, trichostatin A.

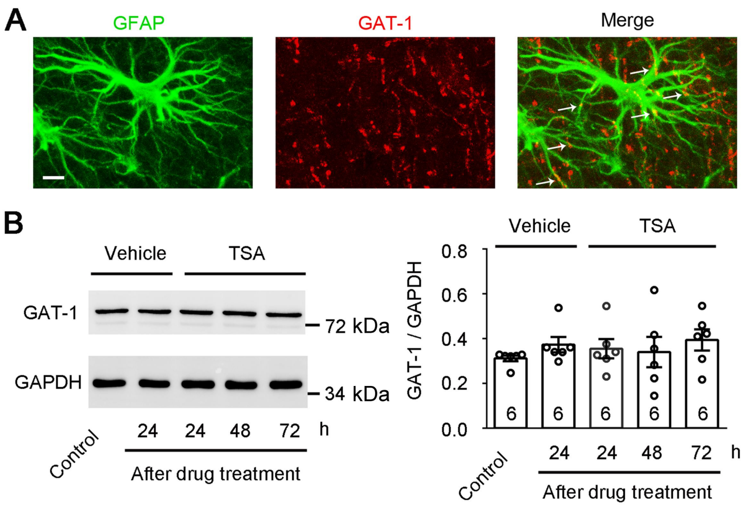

Figure S5. OGD and TSA treatment have no effect on the expression of GAT-1 in astrocytes.

(A) Representative fluorescence image showing GFAP (an astrocyte marker) and GAT-1 in the peri-infarct area. Arrows indicate GAT-1 co-localized with astrocytes. Scale bar, 20 µm. **(B)** Cultured astrocytes were exposed to 3 h of OGD. TSA (0.5 µM) was applied for 24, 48, and 72 h, starting 24 h after OGD. Immunoblots showing the time course of GAT-1 levels in cultured astrocytes after OGD exposure and TSA treatment. Bar graph showing the time course of GAT-1 levels in astrocytes after OGD exposure and TSA treatment. One-way ANOVA followed by post hoc Scheffe test, $F_{(4,25)} = 0.49$. OGD, oxygen glucose deprivation; TSA, trichostatin A.

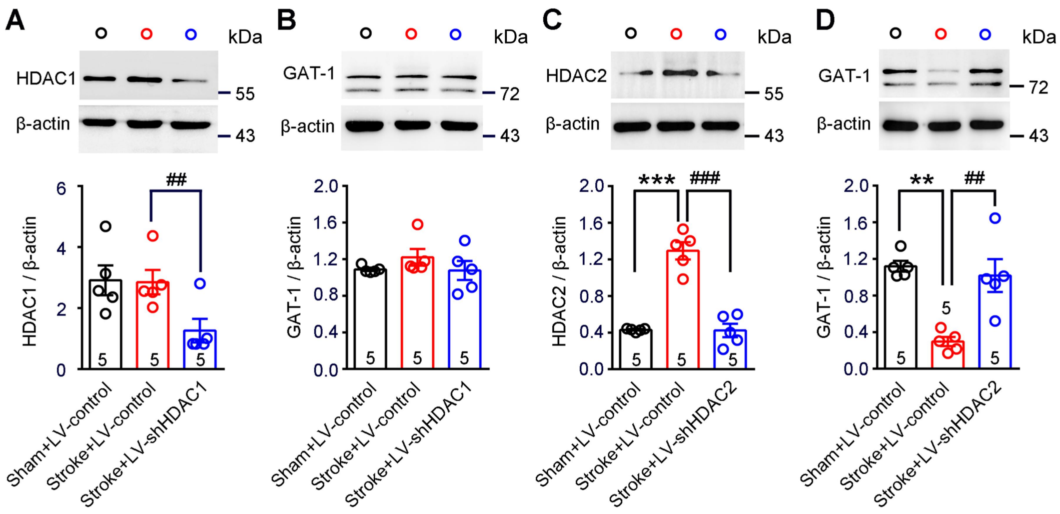

Figure S6. HDAC2 but not HDAC1 negatively regulates GAT-1 expression.

(A) Representative immunoblots and bar graph showing HDAC1 content in the peri-infarct cortex 5 days after stroke. One-way ANOVA followed by post hoc Scheffe test, $F_{(2,12)} = 10.18$, $^{\#\#}p = 0.008$. **(B)** Representative immunoblots and bar graph showing GAT-1 content in the peri-infarct cortex 5 days after stroke. One-way ANOVA followed by post hoc Scheffe test, $F_{(2,12)} = 1$. **(C)** Representative immunoblots and bar graph showing HDAC2 content in the peri-infarct cortex 5 days after stroke. One-way ANOVA followed by post hoc Scheffe test, $F_{(2,12)} = 52.41$, $^{***}p < 0.001$, $^{\#\#\#}p < 0.001$. **(D)** Representative immunoblots and bar graph showing GAT-1 content in the peri-infarct cortex 5 days after stroke. One-way ANOVA followed by post hoc Scheffe test, $F_{(2,12)} = 15.75$, $^{**}p = 0.001$, $^{\#\#}p = 0.003$. HDAC, histone deacetylase.

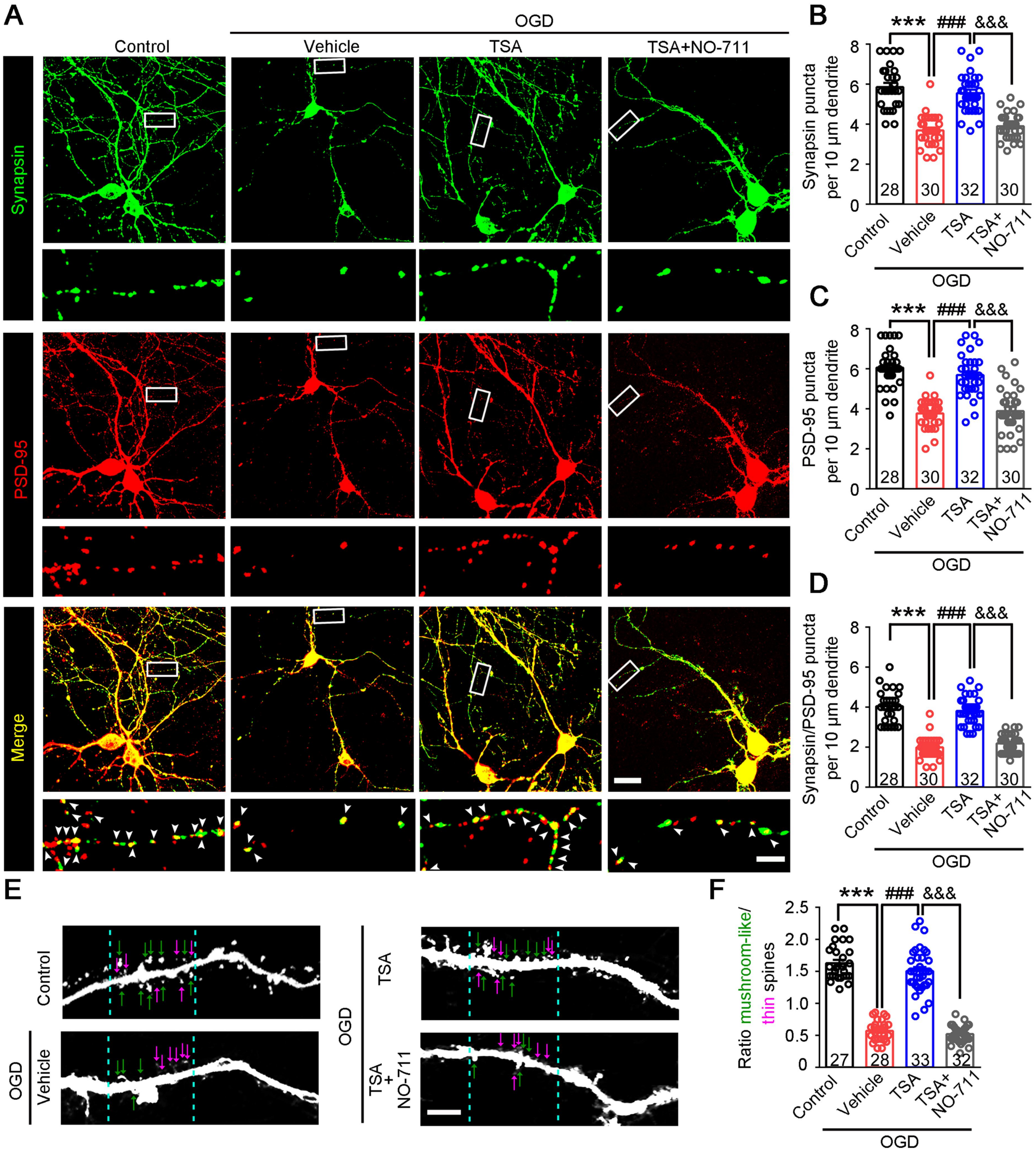

Figure S7. GAT-1 is critical for the TSA-mediated increase in dendritic spine density and synaptogenesis after hypoxia.

(A) Primary cultured mouse cortical neurons were exposed to 3 h of OGD. TSA (0.5 μM) or a mixture of TSA (0.5 μM) and NO-711 (10 μM) was applied for 24 h, starting 24 h after OGD. Representative fluorescence image showing synapsin and PSD-95 in primary cultured mouse cortical neurons after OGD. Each lower panel is a magnified

selected area from the upper iamge showing dendritic spines. Arrows indicate synapsin / PSD-95 double-positive puncta. Upper scale bars, 20 μm. Lower scale bars, 5 μm. **(B)** Bar graph showing the average density of synapsin puncta in dendritic segments of primary cortical neurons of the indicated groups. One-way ANOVA followed by post hoc Scheffe test, $F_{(3,116)} = 46.37$, $^{***}p < 0.001$, $^{\#\#\#}p < 0.001$, $^{\&\&\&}p < 0.001$. n indicates the number of dendrites from 3 independent experiments. **(C)** Bar graph showing the average density of synapsin puncta in dendritic segments of primary cortical neurons with the indicated treatments. One-way ANOVA followed by post hoc Scheffe test, $F_{(3,116)} = 40.74$, $^{***}p < 0.001$, $^{\#\#\#}p < 0.001$, $^{\&\&\&}p < 0.001$. **(D)** Bar graph showing the average density of synapsin / PSD-95 colocalized puncta in dendritic segments of primary cortical neurons with the indicated treatments. One-way ANOVA followed by post hoc Scheffe test, $F_{(3,116)} = 82.06$, $^{***}p < 0.001$, $^{\#\#\#}p < 0.001$, $^{\&\&\&}p < 0.001$. **(E)** Representative images of primary cultured cortical neurons infected with LV-GFP showing the morphology at 48 h after OGD. Scale bar, 5 μm. **(F)** Bar graph showing the ratio of mushroom-like spines versus thin spines in the indicated groups. One-way ANOVA followed by post hoc Scheffe test, $F_{(3,116)} = 170.22$, $^{***}p < 0.001$, $^{\#\#\#}p < 0.001$, $^{\&\&\&}p < 0.001$. n indicates the number of dendrites from 3 independent experiments. OGD, oxygen glucose deprivation; TSA, trichostatin A.

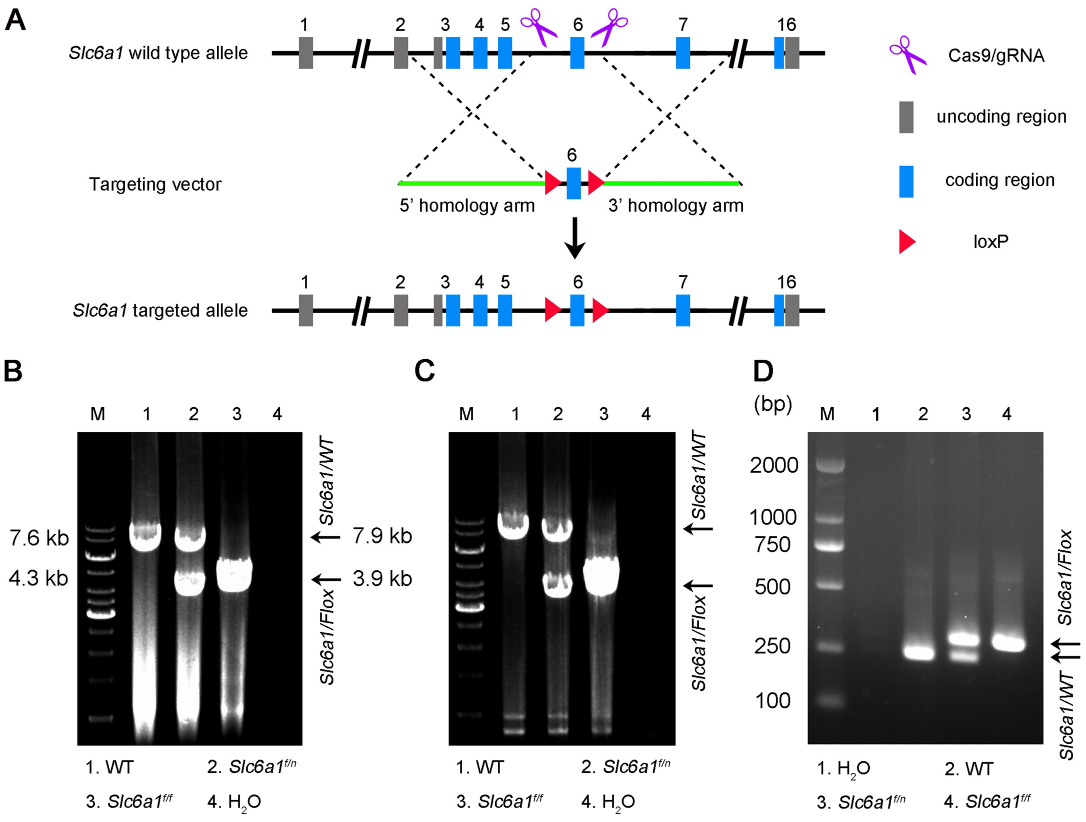

Figure S8. Generation of GAT-1$^{flox/flox}$ mice.

(A) Schematic illustration of the *Slc6a1* gene, targeting vector and the *Slc6a1* floxed locus. The *Slc6a1* gene has 16 exons, and exon 6 was floxed to generate GAT-1$^{flox/flox}$ mice. **(B)** ES clones with 5′ arm homologous recombination were confirmed by PCR verification with a 4.3 kb band, while wild type generated a 7.6 kb band. **(C)** ES clones with 3′ arm homologous recombination were confirmed by PCR verification with a 3.9 kb band, while wild type generated a 7.9 kb band. **(D)** Genotyping of GAT-1$^{flox/flox}$ mice. DNA was isolated for PCR with two primer pairs. The GAT-1 primers generated a 304 bp product in the *loxP*-flanked allele or a 246 bp product in the wild-type allele. M, marker; PCR, polymerase chain reaction.

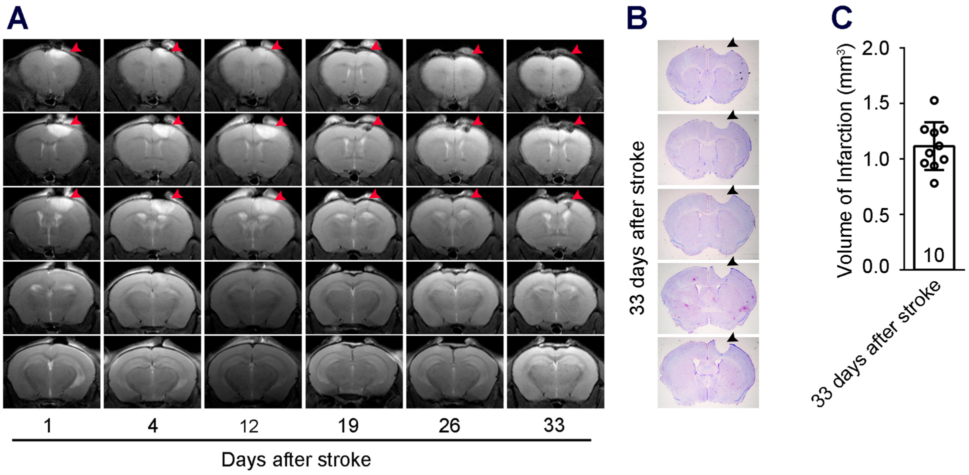

Figure S9. Photothrombotic stroke model induces long-lasting motor cortex injury in adult mice.

(A) Time course of representative T2-TurboRARE MRI of photothrombotic stroke mice. Arrows indicate the injured areas. **(B)** Representative Nissl-stained sections on day 33 after stroke. Arrows indicate the injured areas. **(C)** Bar graph showing stroke volume.

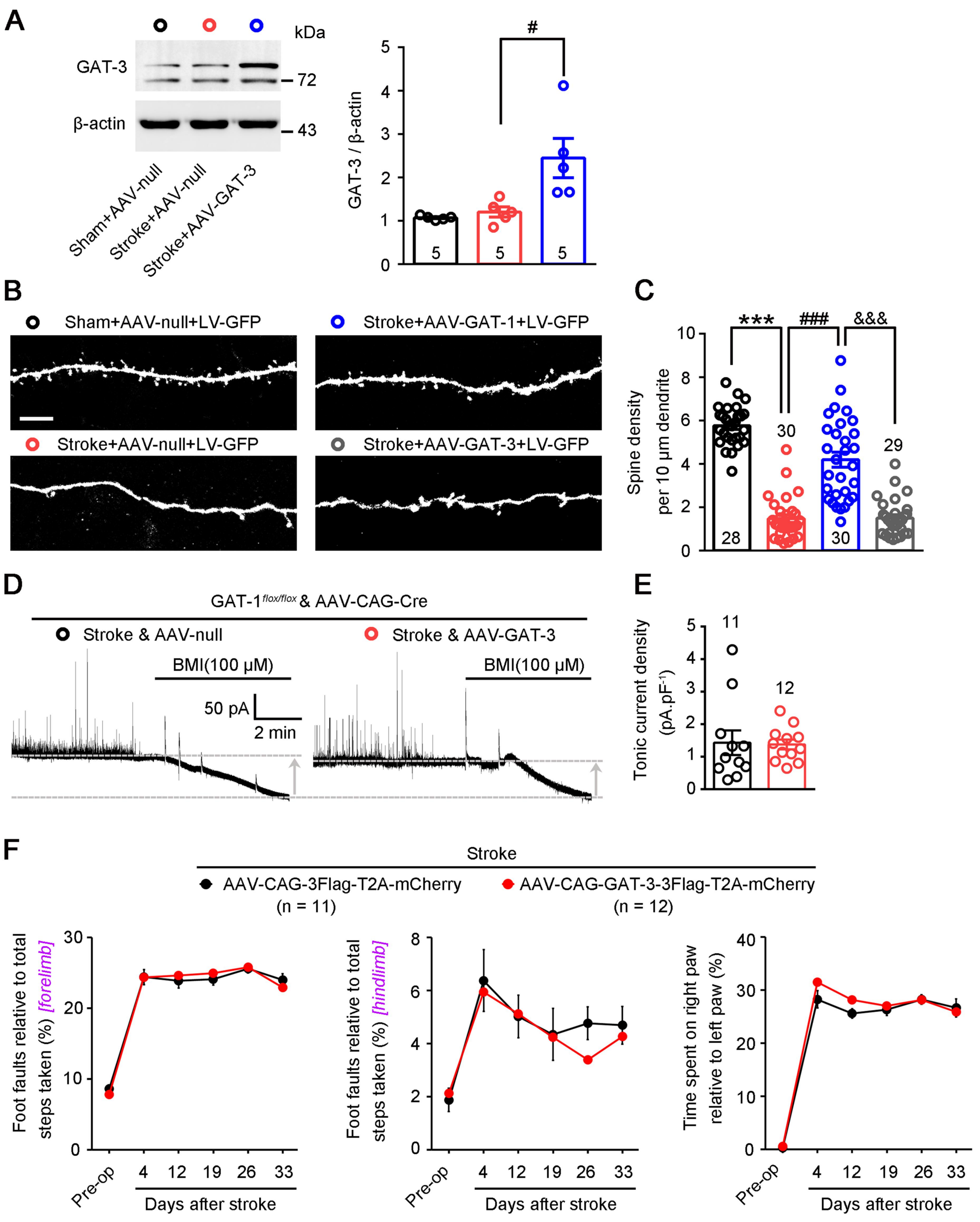

Figure S10. GAT-1 but not GAT-3 overexpression promotes stroke recovery.

(A) GAT-3 level in the peri-infarct cortex 8 days after AAV-CAG-3Flag-T2A-mcherry (AAV-null) or AAV-CAG-GAT-3-3Flag-T2A-mcherry (AAV-GAT-3) infection. One-way ANOVA followed by post hoc Scheffe test, $F_{(2,12)} = 7.87$, $^{\#}p = 0.023$. **(B)** Representative images showing the dendritic spines of peri-infarct neurons infected

with AAV-null, AAV-GAT-1 or AAV-GAT-3. For spine density analysis, only apical dendrites of layer 5 pyramidal neurons in the peri-infarct cortex (within 400 μm from the infarct) were included. Scale bar, 5 μm. **(C)** Bar graph showing spine densities of the indicated group. One-way ANOVA followed by post hoc Scheffe test, $F_{(3,113)}$ = 83.71, $^{***}p < 0.001$, $^{\#\#\#}p < 0.001$, $^{\&\&\&}p < 0.001$. n indicates the number of dendrites from 3 independent experiments. **(D)** Representative traces showing tonic inhibitory currents recorded from layer 5 pyramidal neurons in AAV-null- or AAV-GAT-3-infected GAT-1 knockdown mice. **(E)** Bar graph showing tonic current density from layer 5 pyramidal neurons in AAV-null- or AAV-GAT-3-infected GAT-1 knockdown mice. Two-tailed *t*-test, $F_{(1,21)}$ = 0.02. **(F)** Left, foot faults of the left forelimb in the grid-walking task. Middle, foot faults of the left hindlimb in the grid-walking task. Right, forelimb symmetry in the cylinder task. Two-way repeated-measures ANOVA followed by post hoc Bonferroni test. Left, $F_{(1,22)}$ = 0.001. Middle, $F_{(1,22)}$ = 0.353. Right, $F_{(1,22)}$ = 1.537. AAV, adeno-associated virus; BMI, bicuculline methiodide.
